# Development of Eco-Friendly Hydrogels Loaded with Stoichiometric and Calcium-Deficient Hydroxyapatites for Sustainable Agriculture

**DOI:** 10.1021/acsami.6c03612

**Published:** 2026-04-28

**Authors:** Ganna Yanovska, Olena Goncharuk, Dmytro Honcharuk, Nataliia Guzenko, Ewa Skwarek, Katarzyna Szewczuk-Karpisz

**Affiliations:** † 199348 Institute of Agrophysics, Polish Academy of Sciences, Doświadczalna 4, Lublin 20-290, Poland; ‡ Sumy State University, Kharkivska Str., 2 Sumy 40007, Ukraine; § F.D. Ovcharenko Institute of Biocolloidal Chemistry, NAS of Ukraine, 42 Acad. Vernadskoho Avenue, Kyiv 03142, Ukraine; ∥ Taras Shevchenko National University Academician, Glushkov Avenue, 4, Kyiv 03680, Ukraine; ⊥ Chuiko Institute of Surface Chemistry NAS of Ukraine 17, General Naumov’s Street, Kyiv 03164, Ukraine; # Maria Curie-Skłodowska University, M. Curie-Skłodowskiej Sq. 5, Lublin 20-031, Poland

**Keywords:** alginate hydrogels, hydroxyapatite, calcium-deficient hydroxyapatite, sorption, sustainable agriculture

## Abstract

This study presents the development and characterization of eco-friendly hydrogel composites based on sodium alginate (SA) and two types of hydroxyapatite—stoichiometric (HA) and calcium-deficient (CDHA)—for potential agricultural applications, including soil conditioning and cadmium ion immobilization. The hydrogels were synthesized with varying concentrations of calcium chloride (0.25–0.5%) as a cross-linking agent and different HA/CDHA loadings. Comprehensive physicochemical analyses (X-ray diffractometer, FTIR, scanning electron microscopy/TEM, N_2_ adsorption/desorption) confirmed the successful incorporation of mineral fillers into the alginate matrix and revealed structural differences between HA- and CDHA-filled composites. Swelling studies demonstrated that both filler type and cross-linking density strongly affected water uptake and diffusion mechanisms. HA-filled hydrogels exhibited polymer relaxation-dominated swelling at low cross-linker concentrations, while CDHA-filled systems were governed primarily by diffusion. For Alg/HA gels with a filler concentration of 20%, 33.3%, and 50%, the equilibrium swelling degrees (*Q*_∞_) were 83.02 g/g, 59.36 g/g, and 31.60 g/g, respectively, while the corresponding values for CDHA-filled gels were 80.22 g/g, 81.62 g/g, and 46.48 g/g. When the cross-linker concentration was increased to 0.5 wt %, a substantial reduction in *Q*_∞_ was observed across all composite samples. Sorption experiments showed that the composites effectively sorbed cadmium ions (Cd^2+^) with maximum capacities of 157.7 mg/g (HA-filled) and 190.6 mg/g (CDHA-filled). Experimental data were better described by the Langmuir model. The specific surface area of CDHA was significantly higher than that of stoichiometric HA and equaled 123.6 m^2^/g, whereas the total pore volume of both hydroxyapatites remained similar. The mesopore surface area of CDHA was 102.8 m^2^/g, indicating better sorption properties than in the case of HA, for which this parameter was 57.7 m^2^/g. Biosafety tests using *Pisum sativum* and *Lepidium sativum* seeds confirmed the nontoxic nature of the materials. The composite hydrogels stimulated root elongation, and its high swelling degree supported water retention in soil. Owing to the ability to sorb Cd^2+^ ions, they promoted immobilization of heavy metals, limiting transfer of toxic species to crops. The applied hydroxyapatite improved soil fertility through the supply of mineral components. All these effects indicated that alginate–hydroxyapatite hydrogels can serve as multifunctional materials supporting more sustainable soil management in agriculture.

## Graphical abstract

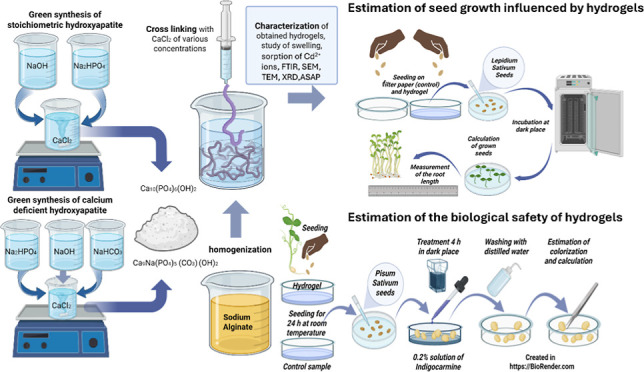

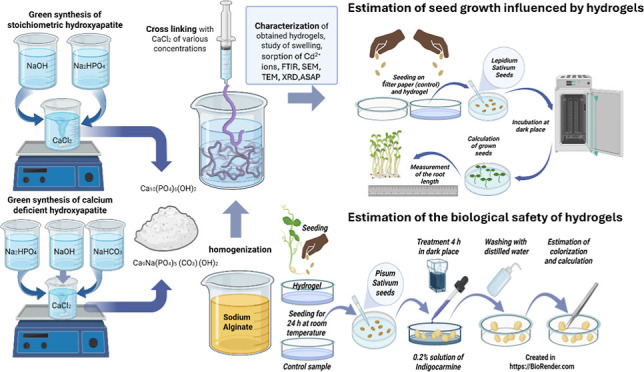

## 1 Introduction

The European Union has outlined an ambitious pathway for soil protection and restoration through the EU Soil Strategy for 2030,^1,2^ which establishes a strategic framework and targeted action allowing the soils to maintain the ability to perform key functions (food production, carbon storage, biodiversity, water retention). In alignment with the broader Zero Pollution Action Plan for 2050,^3^ it is also a priority to achieve the levels of air, water, and soil pollution so low that they are not considered harmful to humans or the environment. Current remediation strategies are often limited by insufficient sorption efficiency and lack of multifunctionality, necessitating advanced materials that integrate pollutant immobilization with improving water-retaining properties for soil conditioning.

Among various candidates, alginate-based hydrogels have attracted considerable attention for agricultural and environmental applications due to their high water retention capacity as well as the ability to control the release of nutrients and promote plant growth.^4^ Long-term soil moisture and more efficient nutrient utilization are essential for maintaining crop productivity while reducing water consumption and environmental losses. Sodium alginate is a biodegradable and environmentally friendly substance derived from seaweed, which exhibits strong affinity for heavy metals due to its chelating properties and the presence of reactive functional groups.^5^ It can effectively bind cadmium (Cd), which is often introduced into water and soil as a result of human activity and poses a threat to the environment. Cd has no physiological functions and can damage various organs like kidneys, liver, and circulatory system.^6^ Its concentrations in leachates from municipal landfills in Europe were even 2700 μg/L.^7^ The Cd binding to the alginate carboxylic groups can effectively limit its bioavailability and penetration into organisms, which is of great importance for remediation processes. By effectively binding water molecules and gradually releasing them, alginate can make soils more resistant to increasingly frequent droughts caused by climate change. Thus, the preparation of hydrogel from exactly this compound can solve many aspects at the same time, i.e., water retention, nutrient supply, and soil conditioning, which will ultimately contribute to more sustainable soil management.

Hydrogels can be made using a variety of fillers that provide a range of water retention properties, sorption of heavy metals from the soil, or gradual release of nutrients.^8^ Hydroxyapatite (HA) can be successfully incorporated into the hydrogels based on sodium alginate or other biopolymers, such as chitosan or gelatin.^9^ As it was mentioned above, alginate is a natural, renewable, and biocompatible polysaccharide,^10^ while HA is a bioapatite closely resembling mineral phases of bone and tooth, and owing to its high phosphorus and calcium content, it can be useful for plants.^11,12^ Their combination yields safe and biodegradable materials. So far, HA was reported to be an effective sorbent,^13^ capable of capturing toxic compounds, pollutants, and heavy metals, and uniquely capable of hosting cations and anions within its crystal lattice without losing chemical stability.^14^ Various modifications and substitutions in HA allowed us to change its surface and sorption properties.^15^ For example, calcium-deficient apatite was considered an effective sorbent for strontium ions.^16^ Most studies suggested that the exchange of heavy metal ions for calcium on hydroxyapatite surfaces was an important sorption mechanism.^17^ Nano-HA was described as an effective crop fertilizer used for the slow release of phosphorus, that is, as a carrier of nutrients in precision agriculture.^18–21^

Composite hydrogels based on alginate and hydroxyapatite have the potential of multifunctional biomaterials that combine the water-retaining capacity of polysaccharide networks with the sorption and ion-exchange properties of inorganic apatites. The interaction mechanisms between alginate and hydroxyapatite can be multifactorial. Carboxylic groups (−COO^–^) of alginate can coordinate calcium ions (Ca^2+^) from both the cross-linker (CaCl_2_) and HA/CDHA particles, thereby stabilizing the “egg-box” structure of the hydrogel.^22^ Hydroxyl (–OH) groups in HA and alginate interact via hydrogen bonds, improving compatibility between phases.^23^ In addition, alginate can chelate calcium ions exposed on HA surfaces, promoting strong interfacial adhesion.^24^

Despite the wide variety of hydrogels currently applied in agriculture, alginate–hydroxyapatite composites have mainly been investigated for biomedical scaffolds,^25,26^ water sorbents,^27^ or soil amendment applications^28,29^ and usually comprise only stoichiometric hydroxyapatite. The incorporation of different apatite phases, particularly the comparison of stoichiometric hydroxyapatite (HA) and calcium-deficient hydroxyapatite (CDHA), into an alginate matrix for the development of multifunctional agricultural hydrogels aimed at cadmium immobilization, water retention, and nutrient management has not yet been systematically investigated.

The alginate–hydroxyapatite combination can have synergistic effects to explain the enhanced sorption, water retention, and biocompatibility compared to pure alginate or HA alone. This approach aligns with the broader development of advanced biopolymer-based functional materials, highlighting the versatility of natural polymers in creating multifunctional applications.^30–32^ In this work, two types of apatites—stoichiometric HA and calcium-deficient CDHA—were synthesized and used as inorganic fillers. Their swelling behavior, sorption capacity, and other physicochemical properties were comparatively evaluated in aqueous systems. The developed alginate–HA materials turned out to be promising for sustainable soil management, enabling both moisture conservation and remediation of metal-contaminated environments.

## 2 Materials and Methods

### 2.1 Materials

Sodium alginate (SA, Glentham Life Science, 99%, prod. Code GE7494, CAS:9005-38-3) was used to form polymer matrix of hydrogels, and calcium chloride anhydrous (CaCl_2_, Chempur, CAS: 10043-52-4) was applied as a cross-linking agent for hydrogel and hydroxyapatite (HA and CDHA) syntheses. Disodium hydrogen phosphate (Na_2_HPO_4_, CAS: 7558-79-4), sodium hydrogen carbonate (NaHCO_3_, CAS:144-55-8), and sodium hydroxide (NaOH, CAS:1310-73-2) were of analytical grade and used as purchased. Double-distilled water was applied as a solvent in all experiments.

For biosafety tests, *Lepidium sativum* was delivered by W. Legutko Przedsiębiorstwo Hodowlano-Nasienne Sp. z o.o., while *Pisum sativum* by TORSEED—Przedsiębiorstwo Nasiennictwa Ogrodniczego i Szkłókarstwa S.A.

### 2.2 Synthesis of Hydrogels

Two types of inorganic fillers, HA and CDHA, were used for the hydrogel preparation. The stoichiometric HA was synthesized according to the following reaction

$${10CaCl}_{2}+{6Na}_{2}{HPO}_{4}+8NaOH\to {Ca}_{10}({PO}_{4}{)}_{6}{\left(OH\right)}_{2}+20NaCl+6H_{2}O$$

The 0.1 M CaCl_2_ solution was mixed with the NaOH solution to adjust pH 12. It was heated under stirring up to 80 °C. The second solution, 0.06 M Na_2_HPO_4_, was added dropwise to a 0.1 M CaCl_2_ solution to obtain hydroxyapatite under stirring, and the mixture was heated to 80 °C for 2 h. The top solution was removed by decantation. After that, the precipitate was washed three times until a pH of approximately 7 was obtained. This allowed us to remove the CaCl_2_ residues. After decantation, the precipitate was filtered and dried to obtain powder.

The calcium-deficient HA was synthesized as described in^33^ according to the reaction

$$9{CaCl}_{2}+5{Na}_{2}{HPO}_{4}+8NaOH+{NaHCO}_{3}\to {Ca}_{9}Na({PO}_{4}{)}_{5}({CO}_{3}{)\left(OH\right)}_{2}+18NaCl+6H_{2}O$$

The CaCl_2_ solution (0.09 M) was prepared in distilled water and heated to 80 °C under constant stirring. Sodium bicarbonate was added to obtain a final concentration of 0.01 M and to introduce carbonate ions into the reaction medium. Next, the 0.05 M NaH_2_PO_4_ solution was prepared and added dropwise to the calcium-containing solution while maintaining the temperature of 80 °C. The reaction was continued for 2 h under vigorous stirring. The pH value was kept above 11 by the addition of the NaOH solution and then adjusted to 9 after completion of the reaction. The suspension was aged for 24 h at room temperature, after which the supernatant was removed by decantation. The precipitate was washed three times with deionized water until a neutral pH was reached and used further in the form of a slurry.^33^ This washing allowed us to remove the CaCl_2_ residues.

For hydrogel preparation, required amounts (2.5 g, 5 g, 10 g) of two types of inorganic fillers, HA and CDHA, were mixed with dry 2% sodium alginate in the ratios described in Table 1. The concentration of SA was taken according to the previous investigations,^6^ and the mixtures of HA/Alg or CDHA/Alg were dissolved in the required amount of water according to Table 1.

**Table 1:** Parameters of Hydrogel Synthesis

	sample composition			
sample number	type (amount) of hydroxyapatite	*m*_Alg_ (g)^a^	*V*_H_2_O_ (mL)	CaCl_2_ conc. (%)
1	HA	–	–	
2	CDHA	–	–	
3	HA (2.5 g)	10	500	0.25
4	HA (5 g)	10	500	0.25
5	HA (10 g)	10	500	0.25
6	HA (2.5 g)	10	500	0.30
7	HA (5 g)	10	500	0.30
8	HA (10 g)	10	500	0.30
9	HA (2.5 g)	10	500	0.50
10	HA (5 g)	10	500	0.50
11	HA (10 g)	10	500	0.50
12	CDHA (2.5 g)	10	500	0.25
13	CDHA (2.5 g)	10	500	0.30
14	CDHA (2.5 g)	10	500	0.50
15	CDHA (5 g)	10	500	0.25
16	CDHA (5 g)	10	500	0.30
17	CDHA (5 g)	10	500	0.50
18	CDHA (10 g)	10	500	0.25
19	CDHA (10 g)	10	500	0.30
20	CDHA (10 g)	10	500	0.50

a Mass of 2% sodium alginate (Alg).

The HA/Alg and CDHA/Alg mixtures were extruded through a syringe into the solution of a CaCl_2_ cross-linking agent_,_ with various concentrations of 0.25%, 0.3%, and 0.5%. The obtained solutions of polysaccharides and fillers were kept for 1 h until full cross-linking took place. The obtained hydrogels were washed with distilled water and dried at 60 °C. After drying, the obtained material was ground and used for the following investigations. As a result, the hydrogels had the grounded form.

The scheme of the hydrogel synthesis is presented in Figure 1.

**Figure 1:**
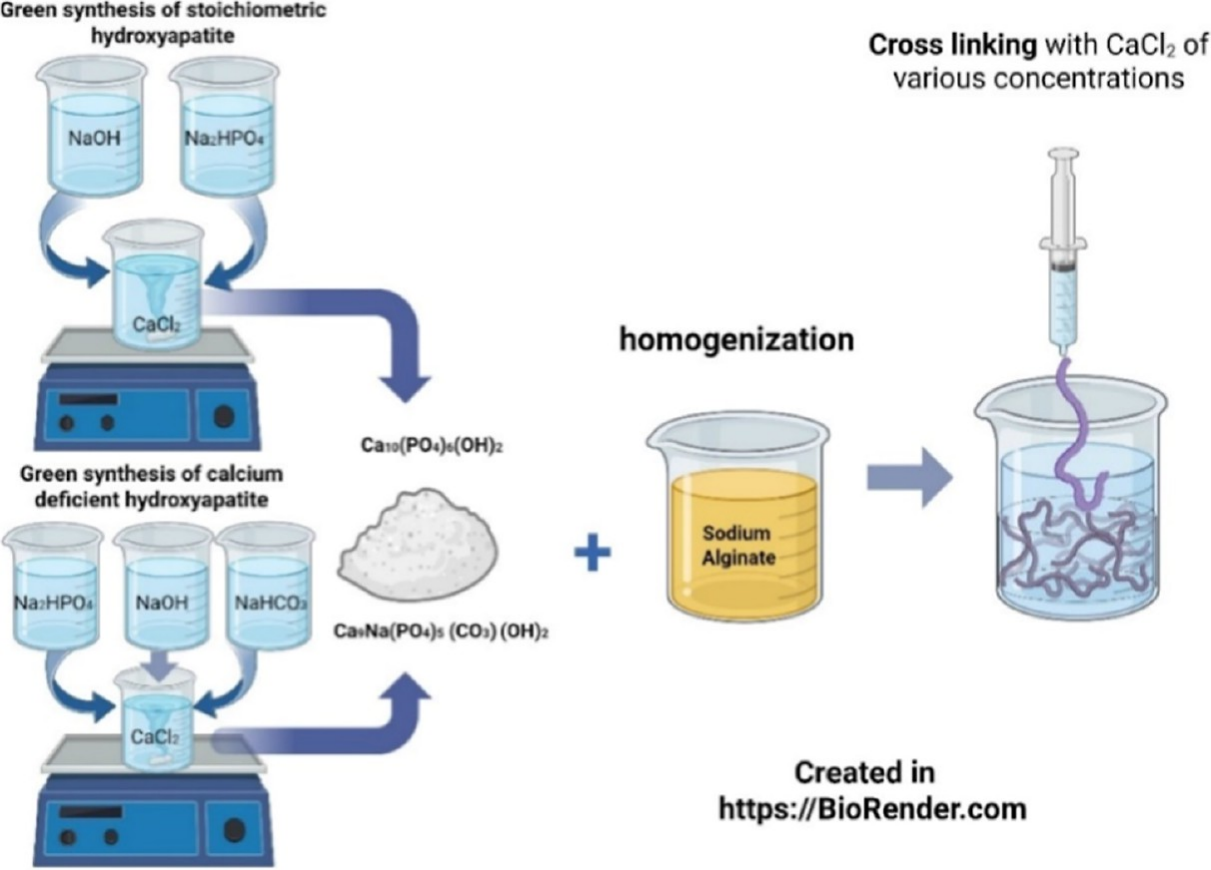
Scheme of the hydrogel synthesis.

### 2.3 Inorganic Filler Characterization

#### 2.3.1 X-ray Diffraction

X-ray phase analysis of the samples using an X-ray diffractometer (Empyrean, PANalytical) was performed to confirm the phase composition of two types of hydroxyapatites obtained according to the above-described procedure. The details of X-ray measurements are described in the Supporting Information.

#### 2.3.2 Textural Analysis

To analyze the textural characteristics of HA and CDHA, low-temperature (77.4 K) nitrogen adsorption–desorption isotherms were recorded using an ASAP2405 (Micromeritics Inc.) adsorption analyzer. The values of the specific surface area (SSA, *S*_BET_) were calculated according to the standard BET method.^34^ The total pore volume, *V*_*p*_, was evaluated by converting the volume of adsorbed nitrogen at *p/p*_0_ = 0.98–0.99 (*p* and *p*_0_ denote the equilibrium and saturation pressures of nitrogen at 77.4 K, respectively) to the volume of liquid nitrogen per gram of adsorbent. The nitrogen desorption data were used to compute the pore size distributions (PSD_S_, differential *f*_V_ ∼ d*V*_p_/d*R* and *f*_S_ ∼ d*S*/d*R*) using a self-consistent regularization (SCR) procedure under non-negativity condition (*f*_V_ ≥ 0 at any pore radius *R*) at a fixed regularization parameter α = 0.01 with voids (V) between spherical nonporous nanoparticles packed in random aggregates (V/SCR model).^35^ The differential PSD_S_ for pore volume *f*_V_ ∼ d*V*/d*R*, ∫*f*_V_d*R* ∼ *V*_p_ were recalculated to incremental PSD (IPSD) at Φ_V_(*R*_*i*_) = (*f*_V_(*R*_*i*+1_) + *f*_V_(*R*_*i*_))­(*R*_*i*+1_ – *R*_*i*_)/2 at ∑Φ_V_(*R*_*i*_) = *V*_p_. The *f*_V_ and *f*_S_ functions were also used to calculate contributions of nanopores (*V*_nano_ and *S*_nano_ at 0.35 nm < *R* < 1 nm), mesopores (*V*_meso_ and *S*_meso_ at 1 nm < *R* < 25 nm), and macropores (*V*_macro_ and *S*_macro_ at 25 nm < *R* < 100 nm).

The average pore radius *R*_p,V_ was estimated from the ratio of the total pore volume to the specific surface area, assuming cylindrical pore geometry

1 $$R_{p,V}=\frac{2V_{p}}{S_{BET}}$$

Additionally, the pore size distributions were calculated from nitrogen desorption data using the SCR method, and the average pore radius was also evaluated as a volume-weighted mean from the PSD.

### 2.4 Hydrogel Characterization

#### 2.4.1 Morphology Characterization

High-resolution scanning electron microscopy (SEM) (Quanta 3D FEG, FEI) and transmission electron microscopy (Tecnai, G2 T20 X-TWIN, FEI) were applied for the morphology and microstructure characterization of sodium alginate-based hydrogels and inorganic fillers HA and CDHA. A Tecnai G2 T20 X-TWIN transmission electron microscope (FEI) was operated at an accelerating voltage of 200 kV and a point resolution of ∼0.24 nm. Prior to analysis, freeze-dried samples were dispersed in ethanol and ultrasonicated for 10–15 min to obtain a homogeneous suspension. A drop of the suspension was deposited onto a carbon-coated copper grid and dried at room temperature. Bright-field TEM imaging was used to evaluate the particle size, morphology, and distribution of HA and CDHA within the alginate matrix. The spot size and condenser aperture were adjusted to optimize image contrast and minimize beam damage to the polymer matrix. Selected area electron diffraction patterns were recorded using a selected area aperture with a typical camera length in the range of 100–200 mm to assess the crystallinity of the inorganic phase. High-resolution TEM (HRTEM) analysis was conducted in selected regions to examine lattice fringes and structural ordering of hydroxyapatite.

#### 2.4.2 FTIR Characterization

FTIR spectra of the samples were obtained in the 4000–400 cm^–1^ range using a ThermoNicolet iS10 spectrometer equipped with a diamond ATR (attenuated total reflectance) accessory. Before analysis, the dried samples were thoroughly ground using a mortar and pestle to obtain a uniform powder. The spectra were recorded with a spectral resolution of 4 cm^–1^ by averaging 32 scans.

#### 2.4.3 Swelling Studies

The swelling studies of the synthesized gels were conducted using the weight method at room temperature (22 °C) by measuring the mass of the swollen gels over time as they were exposed to water. The details of the method are described in the SI section.

The swelling degree, *Q*, was determined gravimetrically according to the formula^36^

2 $$Q=\frac{\left(w_{t}-w_{0}\right)}{w_{0}}$$

3 $$Q_{\infty }=\frac{\left(w_{\infty }-w_{0}\right)}{w_{0}}$$

where *w*_*0*_ is the weight of a dried hydrogel sample after preparation. *W*_*t*_ is its weight at the swelling time *t*, and *w*_∞_ is the weight of a fully swollen at an equilibrium state.

To evaluate the mechanism of water diffusion in the synthesized HGs, the Korsmeyer–Peppas model and the following equation were used

4 $$F=M_{t}/M_{\infty }=kt^{n}$$

where *F* denotes the amount of the solvent fraction at time *t*; *M*_*t*_ and *M*_∞_ correspond to the amount of solvent diffused into the gel at time *t* and at time ∞ (in the equilibrium state), respectively; *k* is a constant associated with the structure of the HG network; the swelling exponent *n* is the number that determines the type of diffusion.^37^

#### 2.4.4 Sorption of Cadmium by Hydrogels

The study on sorption of Cd ions on the dried composite materials obtained using inorganic fillers—HA and CDHA (mixed with sodium alginate in the following ratio: 1:1, 1:0.5, and 1:0.25 in dry condition), and polysaccharide—sodium alginate, and three concentrations of cross-linking agents—CaCl_2_ (0.25%, 0.3%, and 0.5%)—was analyzed. The detailed method was described in SI.

The experimental data were fitted to two of the most frequently used isotherm models, the Freundlich and Langmuir ones,^38^ to characterize the amount of sorbed Cd^2+^ ions on the hybrid HA/Alg and CDHA/Alg hydrogels as a function of equilibrium ion concentration as well as the sorption mechanism.

The Langmuir isotherm model assumes monolayer sorption on a uniform surface where all sorption sites are identical, and there is no interaction between the sorbed molecules, and is described by the equation

5 $$q_{e}=\frac{q_{m}K_{L}C_{eq}}{{1+K_{L}C}_{eq}}$$

where *q*_e_ is the amount of Cd^2+^ ions sorbed per unit mass of sorbent at equilibrium (mg/g); *C*_eq_ is the equilibrium concentration of the sorbate in the solution (mg/L); *q*_m_ is the maximum sorption capacity, representing the maximum amount of sorbate that can be sorbed to form a complete monolayer on the sorbent surface (mg/g); and *K*_L_ is the Langmuir constant, related to the affinity of binding sites and the energy of sorption (L/mg).

To calculate the isotherm parameters, the Langmuir equation was converted to a linear equation, and *q*_m_ was determined as the 1/slope and *K*_L_ as 1/(intercept·*q*_m_) from the plot of *C*_e_/*q*_e_ versus *C*_e_.

The Freundlich isotherm model, which describes the sorption of dissolved substances from a liquid on heterogeneous solid surfaces with sorption energy distribution, was calculated based on the equation

6 $$q_{e}=K_{F}{C_{\mathrm{eq}}}^{1/n}$$

where *q*_e_ is the amount of sorbed Cd^2+^ ions (mg/g); *C*_eq_ is the equilibrium concentration of the Cd^2+^ ions in the solution (mg/L); *K*_F_ is the Freundlich constant, which indicates the sorption capacity of the adsorbent for the sorbate ((mg/g)·(L/mg)^1/*n*^); and 1/*n* is the Freundlich sorption intensity or heterogeneity factor (dimensionless).

The sorption parameters 1/*n* were determined as the slope and log *K*_F_ as the intercept from the linear form of the Freundlich equation by plotting log *q*_e_ versus log *C*_eq_.

### 2.5 Characterization of Hydrogel Biosafety

#### 2.5.1 Determination of Biosafety by Nelubov’s Method

To evaluate the potential toxicity of hydrogel composites, the assessment of the viability of pea seed embryos after incubation with the tested hydrogel samples was provided.^39^ Three replicates of 20 seeds per hydrogel sample of pea seeds (*P. sativum*) were prepared for each hydrogel sample. The seeds were placed in contact with the hydrogel and incubated at room temperature for 24 h. After incubation, the seed coats were carefully removed using tweezers. The exposed embryos were transferred into a 0.2% indigo carmine solution and incubated for 4 h at room temperature in the dark. After staining, the embryos were rinsed with distilled water. Viability was assessed based on staining intensity. In a staining test, the embryos were classified according to the intensity and uniformity of staining. Embryos showing homogeneous and intense coloration over the entire surface were considered nonviable or damaged, whereas embryos with weak, uneven, or partial staining were classified as viable. The assessment was performed visually for all samples under identical conditions, and the number of viable embryos was determined by counting unstained or partially stained seeds relative to the total number of tested seeds. The number of viable embryos was counted, and seed viability (%) was calculated using the formula

7 $$\mathrm{viability}=\frac{\mathrm{Number}\;\mathrm{of}\;\mathrm{unstained}\;\mathrm{or}\;\mathrm{partially}\;\mathrm{stained}\;\mathrm{embryos}}{\mathrm{Total}\;\mathrm{number}\;\mathrm{of}\;\mathrm{embryos}}\times 100\%$$

#### 2.5.2 Determination of Lepidium Sativum Index Germination

To assess the potential inhibitory or stimulatory effects of hydrogel materials on seed germination and root elongation in cress seeds (*Lepidium Sativum*), germination rate and root length were analyzed.^40^ Seed germination tests were carried out under controlled laboratory conditions at room temperature (22 ± 2 °C). Three sets of 10 *L. sativum* seeds were placed in moistened filter paper in Petri dishes on top of swollen hydrogels (1 g of hydrogel in 50 mL of water) coated with filter paper. Moisture was maintained by the periodic addition of distilled water to prevent drying of the substrate. Distilled water was used as the control. The seeds were kept under natural light–dark conditions for 72 h from the start of incubation with no additional illumination. Germination and root growth were evaluated after the specified incubation time by counting the number of germinated seeds and measuring the root length using a ruler.

The percentage of germination—relative seed growth (RSG)—and root length—relative root growth (RRG)—were calculated. Germination rate (%) was calculated by the following equation

8 $$\mathrm{germination}\;\mathrm{rate}=\left(\frac{Number\;of\;germinated\;seeds}{10}\right)\times 100\%$$

RSG was calculated as follows:

9 $$\mathrm{RSG}=\left(\frac{N\;\mathrm{grown}\;\mathrm{seeds}\;\mathrm{in}\;hydrogel}{N\;\mathrm{grown}\;\mathrm{seeds}\;in\;control}\right)\times 100\%$$

Relative root growth (RRG) compared to the control was calculated by the following equation

10 $$\mathrm{RRG}\left(\%\right)=\left(\frac{N\;\mathrm{root}\;\mathrm{length}\;\mathrm{in}\;hydrogel}{N\;root\;length\;in\;control}\right)\times 100\%$$

In turn, growth index was calculated as follows

11 $$\mathrm{GI}=\frac{\mathrm{RSG}\times \mathrm{RRG}}{100\%}$$

### 2.6 Statistical Analysis

All measurements were performed three times, and the graphs present mean values. Standard deviations were calculated using Statistica (13.3, StatSoft Inc.). Descriptive analysis (mean ± SD) was used due to the limited number of replicates, without formal statistical hypothesis testing.

## 3 Results and Discussion

### 3.1 Structural Characterization

The X-ray diffraction results indicated that the phase composition of inorganic fillers corresponded to hydroxyapatite (JCPDS card 04-021-1904) (Figure S1).

The textural parameters of CDHA and HA hydroxyapatites are summarized in Table 2, whereas the N_2_ adsorption/desorption isotherms are presented in Figure 2.

**Table 2:** Textural Characteristics of Inorganic Fillers^a^

sample	*S*_BET_ (m^2^/g)	*S*_micro_ (m^2^/g)	*S*_meso_ (m^2^/g)	*S*_macro_ (m^2^/g)	*V*_micro_ (cm^3^/g)	*V*_meso_ (cm^3^/g)	*V*_macro_ (cm^3^/g)	*V*_p_ (cm^3^/g)	*R*_p_,_V_ (nm)
HA	80.6	19.0	57.7	3.9	0.01	0.54	0.06	0.6	16.85
CDHA	123.6	20.5	102.8	0.3	0.01	0.58	0.01	0.6	9.71

a Specific surface area in total (*S*_BET_), micropore surface area (*S*_micro_), mesopore surface area (*S*_meso_), macropore surface area (*S*_macro_), and pore volumes (*V*_p_, *V*_micro_, *V*_meso_, *V*_macro_). Micropores correspond to pore radius *R* < 1 nm, mesopores to 1 nm < *R* < 25 nm, and macropores to *R* > 25 nm. *R*_p,V_ is the average pore radius, which was calculated from the pore size distribution obtained using the SCR method as the volume-weighted mean pore radius.

**Figure 2:**
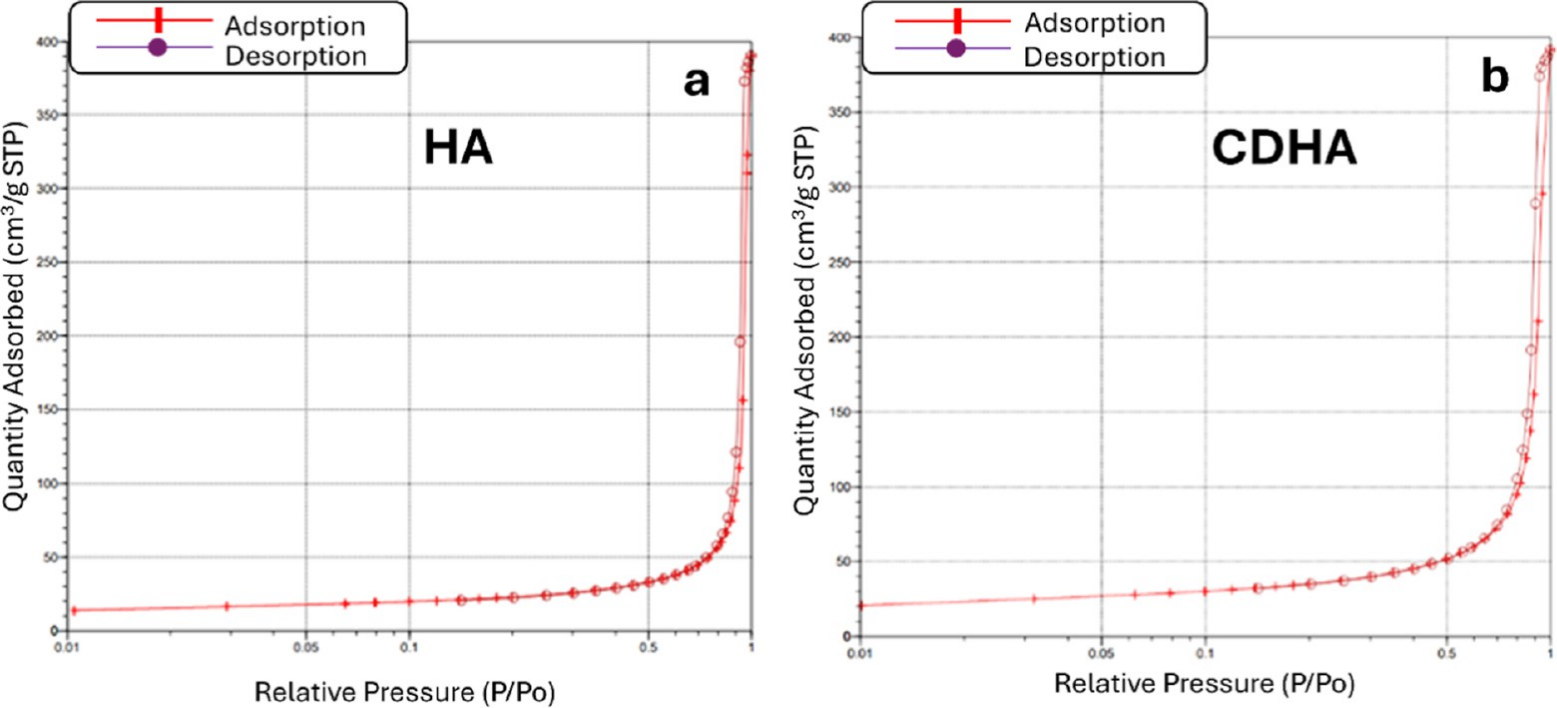
Adsorption/desorption N_2_ isotherms of (a) stoichiometric hydroxyapatite, (b) calcium-deficient hydroxyapatite.

As can be seen from the nitrogen adsorption isotherms of stoichiometric hydroxyapatite and calcium-deficient hydroxyapatite, the adsorption branches exhibited features characteristic of type II isotherms according to the International Union of Pure and Applied Chemistry (IUPAC) classification. At the same time, a narrow hysteresis loop was observed in the intermediate relative pressure range, indicating the presence of mesoporosity associated mainly with interparticle voids formed by aggregates of hydroxyapatite nanoparticles. Such hysteresis reflected capillary condensation occurring in mesopores of mixed geometry (cylindrical, slit-shaped, and ink-bottle type). This combination of type II isotherm features with hysteresis, which was associated with interparticle mesoporosity, was characteristic of powder materials (especially aggregated nanoparticles). According to the calculations of specific surface area and total porosity, based on the obtained N_2_ adsorption/desorption isotherms, the initial stoichiometric hydroxyapatite had a specific surface area (*S*_BET_) of 80.6 m^2^/g and a total pore volume equal to 0.604 cm^3^/g, most of which corresponded to mesopores (Table 2).

The specific surface area of CDHA was significantly higher than that of stoichiometric HA and equaled 123.6 m^2^/g, whereas the total pore volume of both hydroxyapatites remained similar. However, the pore radius was considerably smaller (for CDHA, the average pore radius was 9.71 nm, whereas for HA, it was 16.85 nm). In this case, the mesopore surface area of CDHA was 102.8 m^2^/g, suggesting better sorption properties compared with HA, for which the mesopore surface area was 57.7 m^2^/g. Even though the total pore volume was identical for both materials, the reduction in the average pore radius from HA to CDHA necessitated a much higher density of pores. In a fixed volume, smaller pores resulted in a higher cumulative internal surface area,^41,42^ which explained why the specific surface area of CDHA was nearly double that of HA.

### 3.2 FTIR Analysis

The FTIR analysis was applied to verify the structure and investigate potential interactions occurring between the components of the composite hydrogels. Figure 3 shows the FTIR spectra of sodium alginate, stoichiometric hydroxyapatite, and calcium-deficient hydroxyapatite.

**Figure 3:**
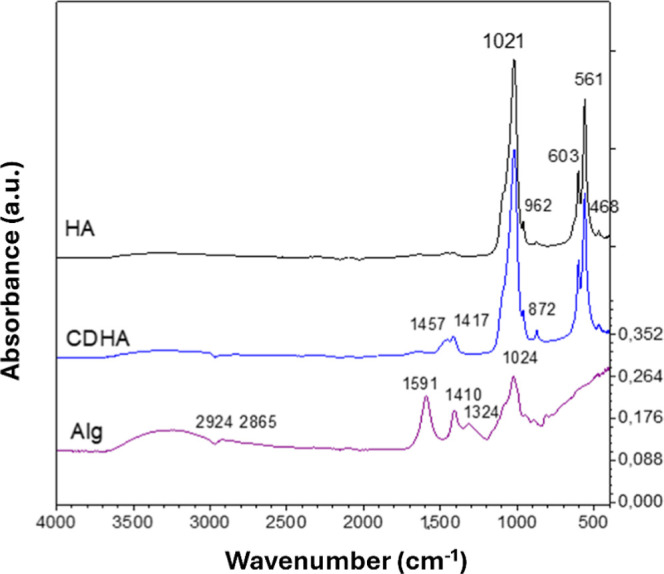
FTIR spectra of stoichiometric hydroxyapatite (HA), calcium-deficient apatite (CDHA), and sodium alginate (Alg).

The spectra of Alg hydrogels showed characteristic peaks of asymmetric and symmetric stretching vibrations of carboxyl groups (–COO^–^) at 1591 and 1408 cm^–1^, respectively. The band at 1024 cm^–1^ corresponded to the C–O stretching vibration of the polysaccharide backbone. The bands at 2924 and 2865 cm^–1^ represented the asymmetric and symmetric stretching vibrations of aliphatic C–H bonds, and the broad band at 3248 cm^–1^ was due to the –OH stretching and adsorbed water molecules.^43^

In the spectra of the hydroxyapatite samples (both stoichiometric and calcium-deficient), characteristic bands of PO_4_^3–^ (ν_1_, ν_2_, ν_3_, ν_4_) were observed with maxima at 468 cm^–1^ (ν_2_ deformation vibration of PO_4_^3–^), 561 and 603 cm^–1^ (ν_4_ deformation vibrations), 962 cm^–1^ (ν_1_ symmetric stretching), and 1021 cm^–1^ (ν_3_ asymmetric stretching).^44,45^ There were also carbonate bands at 872 cm^–1^ (ν_1_ CO_3_^2–^, type B substitution) and 1417 cm^–1^ (ν_3_ asymmetric stretching of CO_3_^2–^). The CDHA spectrum included an intense band at 1457 cm^–1^, assigned to HPO_4_^2–^—hallmark of calcium deficiency. In CDHA, distinct HPO_4_^2–^ bands at 872 and 1457 cm^–1^ further confirmed the defect-rich structure.

In the spectra of the HG composite (Figures 4 and 5), characteristic bands of the individual components remained, and their intensities depended on the concentration of each component. A semiquantitative comparison of the carbonate and phosphate bands was performed using the relative intensity ratio of the carbonate band (1415–1450 cm^–1^) to the phosphate ν_3_ band (∼1030 cm^–1^)­

12 $$R_{{CO}_{3}/{PO}_{4}}=\frac{A_{1415-1450}}{A_{1030-1040}}$$

**Figure 4:**
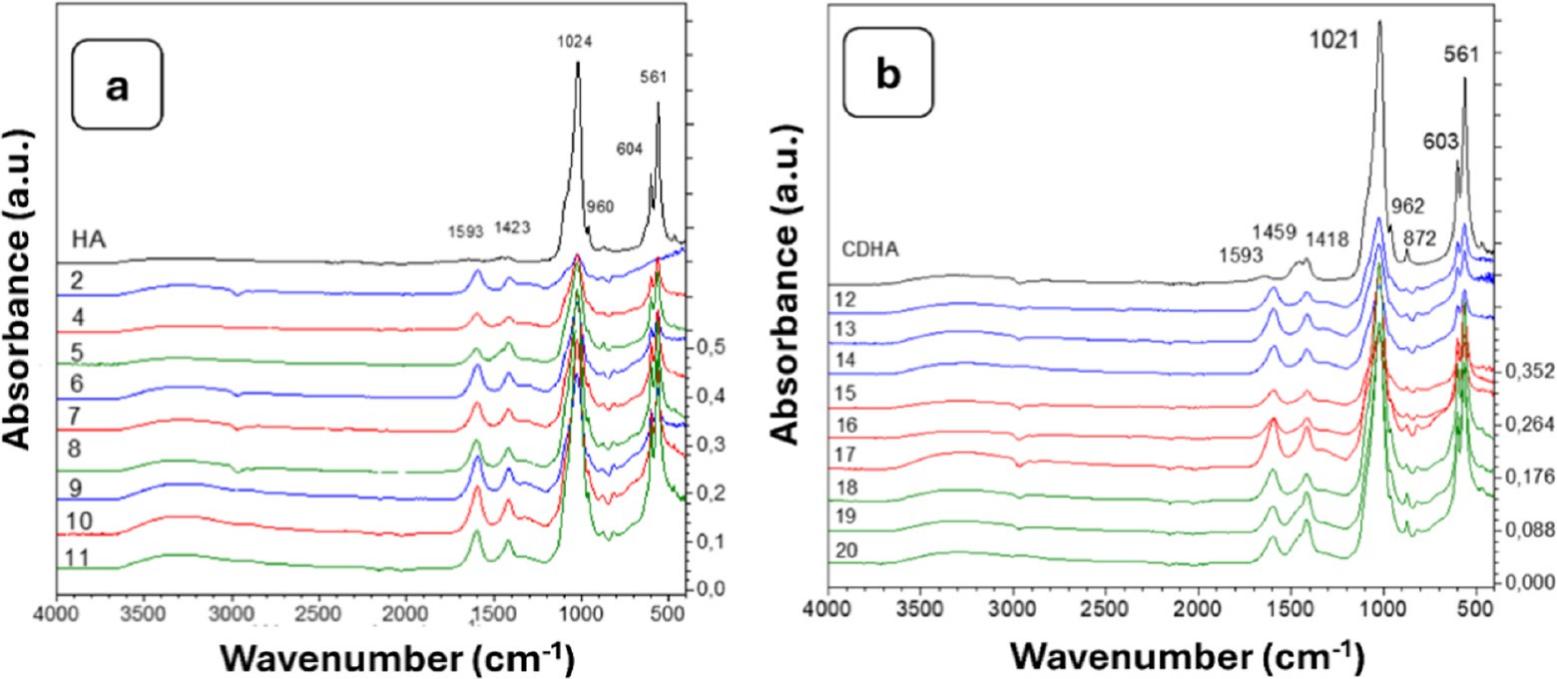
FTIR spectra of stoichiometric hydroxyapatite (a), calcium-deficient hydroxyapatite (b) and their composites with alginate.

**Figure 5:**
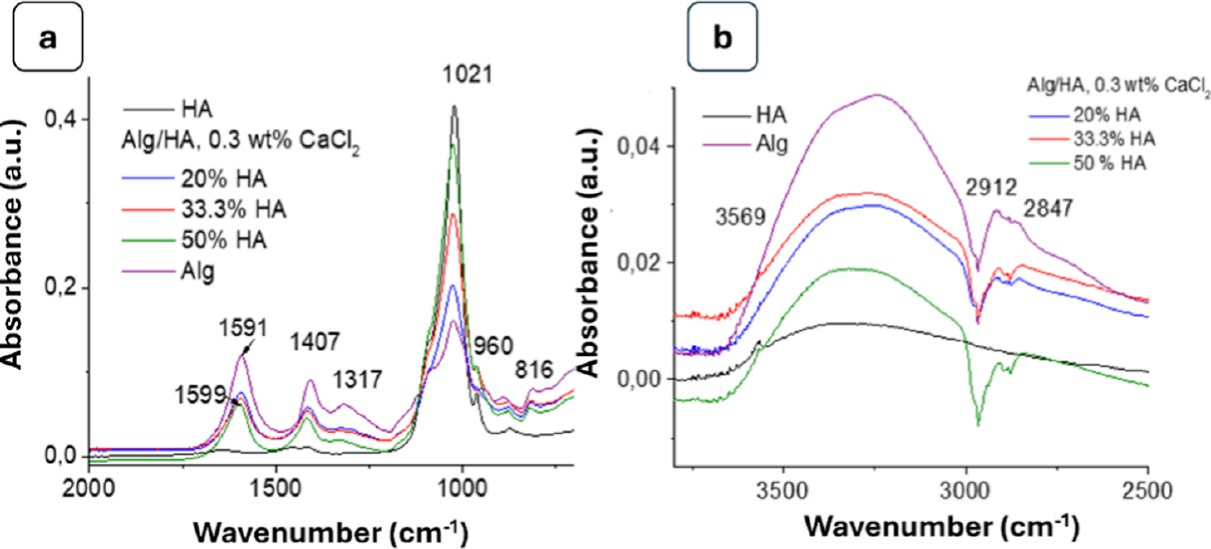
FTIR spectra for HA/Alg hydrogels with various concentrations of HA cross-linked with 0.3% CaCl_2_: (a) at the range 500–2000 cm^–1^, (b) at the range 2500–4000 cm^–1^.

The obtained spectra showed that the carbonate-to-phosphate intensity ratio (*R*_CO_3_/PO_4__), calculated using eq 12, remained relatively low (0.03–0.08 for HA and 0.15–0.25 for CDHA) and did not significantly change after incorporation into the alginate matrix, indicating that the carbonate substitution in the apatite structure was preserved.

A band in the region 872–875 cm^–1^ was observed, which could include overlapping contributions from both B-type carbonate (CO_3_^2–^) and HPO_4_^2–^ groups. In the CDHA samples, this band became more pronounced together with the band at ∼1457 cm^–1^, confirming the presence of HPO_4_^2–^ species associated with calcium deficiency. For CDHA-containing samples, a weak band near 872–875 cm^–1^ attributed to the HPO_4_^2–^ group was also observed. The ratio between the HPO_4_^2–^ band (∼872–875 cm^–1^) and the phosphate ν_1_ band (∼962 cm^–1^) indicated the presence of acid phosphate groups characteristic of CDHA

13 $$R_{{HPO}_{4}/{PO}_{4}}=\frac{A_{875}}{A_{962}}$$

The intensity ratio (*R*_HPO_4_/PO_4__) for CDHA and CDHA-containing composites, calculated using eq 13, was in the range of 0.08–0.20, which corresponded to a moderately defective structure. At low CaCl_2_ concentrations used in the synthesis of composites, competitive binding of Ca^2+^ ions between the filler surface and alginate preserved or slightly increased the relative contribution of the HPO_4_^2–^ groups.

As the hydroxyapatite content increased, the intensities of the phosphate-related bands (962, 1021, 561, 603 cm^–1^) increased proportionally, while intensities of the alginate-related bands (1593–1599, 1408, and 1317 cm^–1^) decreased as the COO^–^ groups became less prominent relative to PO_4_^3–^. The bands at 814 cm^–1^, 1593–1599 cm^–1^, and the broad –OH extension around 3290 cm^–1^ indicated the presence of calcium alginate: 1593–1599 cm^–1^ corresponded to the asymmetric stretching of COO^–^ groups, and 816 cm^–1^ was characteristic of C–O or mannuronic acid fragments. The band at 1024–1026 cm^–1^ appeared broader because it overlapped the PO_4_^3–^ vibrations with C–O from alginate. The spectra of the Alg/HA composites contained classical PO_4_^3–^ and carbonate bands and additional alginate-specific bands at 816, 1408–1415, 1593–1599, 2847, 2912, and 3290 cm^–1^. Compared to the Alg/CDHA composite, the Alg/HA composite lacked the defect-related bands (e.g., 1457 cm^–1^ from HPO_4_^2–^) characteristic of CDHA. Overall, the PO_4_^3–^ structure was preserved in both types of composites.

The lattice –OH band of HA at 3569 cm^–1^ disappeared in both types of composites (filled with HA) (Figure 5b), indicating hydrogen-bonding interactions with the alginate polymer matrix.^46^

As was mentioned above, in all composite samples with HA and CDHA, the characteristic PO_4_^3–^ bands (ν_1_, ν_2_, ν_3_, ν_4_) remained. From alginate to composites, the band identified in the asymmetric COO– stretching mode at 1591 cm^–1^ shifted toward higher wavenumbers (Figure 5a). These blue shifts indicated that there was a chemical interaction between the mineral phase and the organic materials through the chemical bond between Ca^2+^ and the negatively charged carboxyl group in alginate.^47,48^ Thus, the FTIR analysis confirmed interaction between the alginate matrix and hydroxyapatite.

Literature reports suggest several mechanisms, inter alia, alginate COO^–^ groups coordinately bind to Ca^2+^ on the HA or CDHA surfaces, forming chelate complexes.^49^ Additionally, ion exchange between Ca^2+^ in HA/CDHA and Na^+^ or H^+^ in alginate can induce local modifications in HA lattice.^47–50^ Hydrogen bonding also occurs between –OH groups in hydroxyapatite and hydrophilic –OH/–COOH groups in alginate, stabilizing the composite network. These secondary interactions contribute to water retention within the structure, which can increase swelling to some extent, but more importantly, they improve hydrogel cohesion and may enhance sorption by creating a more hydrophilic environment.

Additionally, ion exchange (Ca^2+^ ↔ Na^+^/H^+^) can introduce defects or local distortions in the HA lattice, potentially increasing surface reactivity. This may further improve sorption capacity by generating more active binding sites.

### 3.3 Morphology of Inorganic Fillers and Hydrogels

The crystal size of HA and CDHA was determined to be 8–12 nm. A cylindrical shape for stoichiometric HA and cube shape for CDHA were noticed (Figure 6).

**Figure 6:**
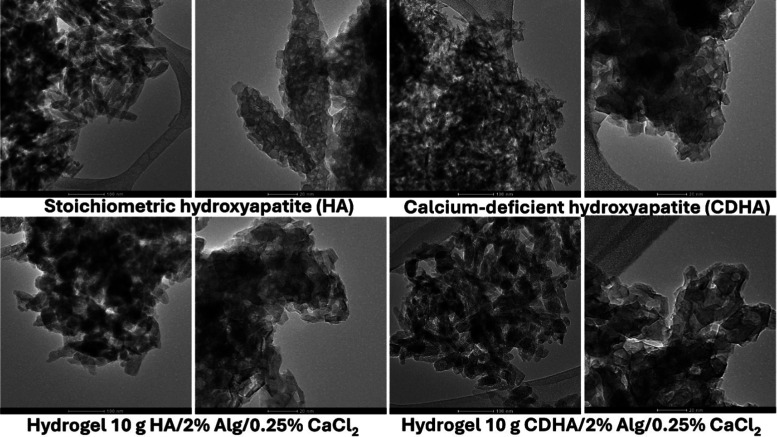
TEM images of HA, CDHA, and hydrogels with various types of inorganic filler (HA and CDHA) synthesized with the CaCl_2_ concentration of 0.25%, i.e., hydrogel 5 and hydrogel 18.

The TEM images revealed clear morphological differences between stoichiometric hydroxyapatite and calcium-deficient hydroxyapatite, both in their pristine form and after incorporation into alginate-based hydrogels. Pristine HA exhibited well-defined nanocrystals with predominantly plate- and needle-like morphologies, forming relatively dense agglomerates with a high degree of crystallinity. In contrast, CDHA particles appeared less ordered, with irregular shapes and lower crystallinity, forming looser and more heterogeneous aggregates. In the case of the hydrogels, the presence of alginate and CaCl_2_ significantly influenced nanoparticle organization. HA incorporated into the hydrogel matrix formed compact and dense aggregates, in which the characteristic morphology of the nanocrystals was still partially preserved but strongly embedded within the polymeric network. Conversely, CDHA in the hydrogel produced less compact, more open, and porous agglomerates, with smaller and less ordered particles compared to HA. These structural differences were consistent with the intrinsic crystallinity and stability of the two hydroxyapatite phases. The denser arrangement of HA nanoparticles within the hydrogel may favor structural reinforcement, whereas the looser and more porous CDHA aggregates could provide higher surface accessibility, which was advantageous for ion exchange and sorption-related applications. The morphology of the hydrogels observed by SEM is presented in Figure S2.

### 3.4 Estimation of Hydrogel Swelling Properties

The effect of HA content and structure on the swelling behavior of composite HG samples, synthesized at different cross-linker concentrations, was analyzed. The HGs were immersed in distilled water with pH values of about 6.5–7.0, and the swelling degrees were measured over time. The swelling behavior of the initial alginate gel without filler was described in detail by Guzenko et al.^51^ The maximum swelling degree of the alginate gels without filler, synthesized with a cross-linking agent concentration of 0.25, 0.3, and 0.5 wt %, was 120 g/g, 68 g/g, and 10 g/g, respectively. The results of the swelling kinetics of the Alg/HA and Alg/CDHA composite HGs, synthesized using 2 wt % alginate and various concentrations of CaCl_2_ in the range from 0.25 to 0.5 wt %, are shown in Figure 7a,b. Figure 7c,d also shows the equilibrium swelling degrees, *Q*_∞_, for the studied samples of Alg, Alg/HA, and Alg/CDHA calculated using eq 2.

**Figure 7:**
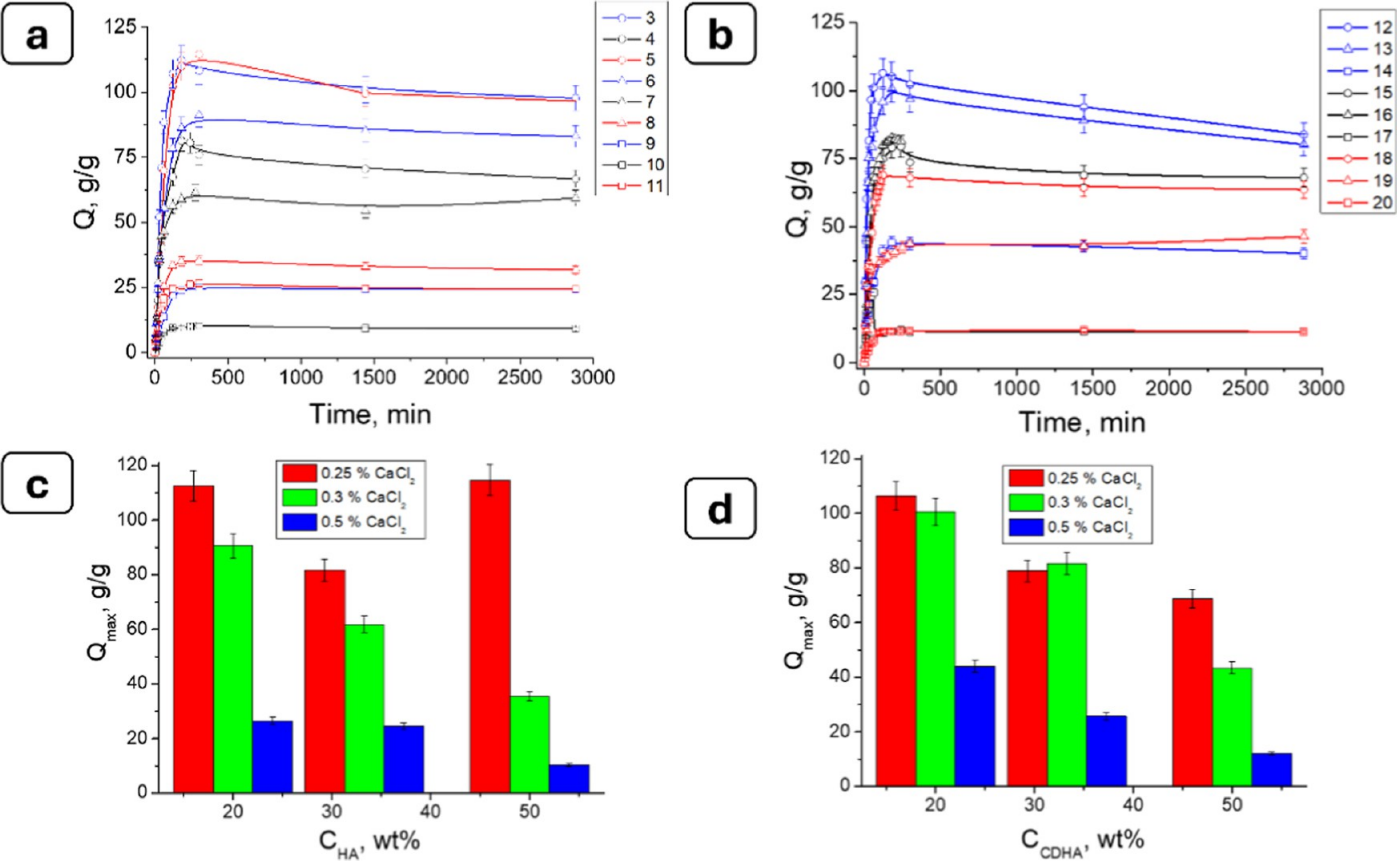
Dependence of swelling of alginate hydrogel samples filled with (a) HA and (b) CDHA on the contact time with distilled water as well as dependence of the maximal degree of swelling on the concentration of the filler (c) HA and (d) CDHA. Data are presented as mean ± SD (*n* = 3).

The swelling kinetic curves showed that the most intense water uptake occurred during the first 4–6 h after placing the samples in aqueous solutions, and this process continued mainly up to 24 h for most studied samples (Figure 7a,b). In the range of CaCl_2_ concentrations from 0.25 to 0.5 wt %, the swelling behavior demonstrated a nonmonotonic dependence on the cross-linker concentration, reflecting changes in the effective cross-linking density of the Alg/HA and Alg/CDHA composite hydrogels. The obtained results agreed with those obtained for other inorganic fillers: laponite and montmorillonite in the alginate-based hydrogel.^51^

The equilibrium swelling degree depended on the cross-linker concentration used in the HG synthesis procedure as well as on the HA filler content (Figure 7c,d). In general, the equilibrium swelling degree *Q*_∞_ tended to decrease with increasing cross-linker concentration and, in most cases, with increasing filler content agreed with previous works.^8,51^ However, at low CaCl_2_ concentration (0.25 wt %), deviations from this trend were observed. For example, in HA-filled hydrogels, the *Q*_∞_ value increased again to 50% filler loading, which may indicate partial disruption of the polymer network due to excessive filler content and reduced efficiency of Ca^2+^ cross-linking. The equilibrium *Q*_∞_ values for the Alg filled with 20%, 33.3%, and 50% HA (0.25 wt % CaCl_2_) samples were 97.79 g/g, 66.67 g/g, and 99.53 g/g and for HGs filled with 20%, 33.3%, and 50%, CDHA were 83.90 g/g, 68.07g/g, and 63.63 g/g, respectively.

At a CaCl_2_ concentration of 0.3 wt %, the hydrogels containing 20%, 33.3%, and 50% of either stoichiometric (HA) or calcium-deficient hydroxyapatite (CDHA) exhibited elastic and resilient structures. These composites retained their shape and did not collapse under gentle manual compression applied using tweezers during handling, even after >200 h of water immersion.

For Alg/HA gels with filler concentrations 20%, 33.3%, and 50%, the equilibrium swelling degrees were 83.02 g/g, 59.36 g/g, and 31.60 g/g, respectively, while the corresponding values for CDHA-filled gels were 80.22 g/g, 81.62 g/g, and 46.48 g/g. When the cross-linker concentration was increased to 0.5 wt %, a substantial reduction in *Q*_∞_ was observed across all composite samples. In the case of HA-filled hydrogels, the *Q*_∞_ values decreased to 24.21 g/g, 9.14 g/g, and 24.29 g/g for samples with filler concentrations 20%, 33.3%, and 50%, respectively, whereas for CDHA-filled gels, they were equal to 40.10 g/g, 11.40 g/g, and 11.30 g/g.

As the presented data showed, the type of filler influenced the swelling behavior in addition to the concentration factor, but this effect was complex. HA had a lower specific surface area (80.56 m^2^/g), implying a reduced active surface for interaction with the gel matrix and Ca^2+^ ions. The relatively high degree of swelling at a filler content of 50% could be explained by impaired structural organization due to filler oversaturation, which lowered the efficiency of Ca^2+^ cross-linking with alginate. Such results agreed with previously obtained data for other inorganic fillers.^51^

CDHA had a higher specific surface area (124.74 m^2^/g) and offered more sites for the interaction with both Ca^2+^ ions and alginate matrix. This could promote more effective physical cross-linking or Ca^2+^ sorption on the HA surface, competing with alginate for available ions. In this case, the gel formation may be less efficient, which explained the higher *Q*_∞_ values observed at low CaCl_2_ concentrations. For instance, at 0.3 wt % CaCl_2_ and 33.3% CDHA, *Q*_∞_ reached approximately 81.62 g/g, significantly higher than the corresponding HA sample (59.36 g/g). At 0.5% CaCl_2_, *Q*_∞_ again decreased sharply, consistent with the effect of excessive ionic strength, which compressed the gel structure and reduced the free volume available for water uptake. The influence of the CaCl_2_ concentration on swelling can be explained by two different mechanisms that dominated at different cross-linker levels.

At low CaCl_2_ concentrations (≈0.25 wt %), part of the Ca^2+^ ions may be adsorbed on the surface of hydroxyapatite particles or involved in competitive interactions with the filler surface. This reduced the effective number of ions available for alginate cross-linking, leading to a looser polymer network and higher swelling degrees.

At higher CaCl_2_ concentrations (0.3–0.5 wt %), the availability of Ca^2+^ ions became sufficient to form a denser egg-box cross-linked alginate network. In addition, the higher ionic strength screened electrostatic repulsion between polymer chains, resulting in a more compact structure and consequently lower equilibrium swelling.

Overall, the trends in the influence of both cross-linker concentration and filler content on swelling were similar for the two types of hydroxyapatite.

The detailed description of swelling mechanisms is presented in SI, Tables S1 and S2.

Low Ca^2+^ ion concentrations resulted in the formation of a weakly cross-linked network, which provided larger swelling. Elevated Ca^2+^ concentrations caused a more compact cross-linked network, diminishing the swelling capacity of HGs.^52^ Thus, in all other cases, increasing the cross-linker concentration yielded stable HGs that absorbed and retained water within their structure without being destroyed or dissolved under experimental conditions, but a significant decrease in the *Q*_∞_ value with the growth cross-linker content was observed especially above 0.5 wt % (Figure 7). The reduction in the swelling of composite HGs containing HA can be due to the HA occupying voids within the HG matrix, diminishing the likelihood of water molecules infiltrating the HG network. In addition, the Alg content per unit dry mass decreased in Alg HGs filled with HA.

It can be summarized that the cross-linking agent concentration below 0.25 wt % was insufficient to form a strong and stable HG network for the samples, as the original Alg HG and Alg/HA composites showed themselves to be more stable under these conditions. The cross-linking agent concentration above 0.5 wt % led to a significant drop in the swelling degree of the studied materials.

The transition from polymer relaxation-controlled swelling to diffusion-dominated behavior with the increasing CaCl_2_ concentration has important implications for water management in agricultural systems. At lower cross-linker concentrations, where swelling is governed by polymer chain relaxation, the hydrogel network is more flexible and less cross-linked. This allows the material to absorb large amounts of water and expand significantly. In soil, such hydrogels act as high-capacity water reservoirs, rapidly taking up water during irrigation or rainfall. However, because their network is less constrained, water is also released relatively quickly. This makes them suitable for short-term water buffering but less effective for prolonged moisture retention. In contrast, at higher CaCl_2_ concentrations, the network becomes more tightly cross-linked, and swelling is mainly controlled by diffusion. The presence of HA or CDHA reinforcing the structure reduces excessive swelling (due to additional cross-linking interactions), improves water retention stability, and provides nutrient-related benefits (e.g., phosphate release), making the system multifunctional.

In addition to the equilibrium degree of swelling, the swelling mechanism also changed with the increasing cross-linking agent concentration. To study the mechanism, the data on swelling kinetics according to eq 3 were used.

In general, the swelling behavior of high-molecular-weight alginate hydrogels (Alg HGs) is governed by a balance between the osmotic pressure driving water uptake into the gel and the elastic restoring forces of the cross-linked network that resist further expansion. As a result, such hydrogels typically reach an equilibrium swelling state when these forces are balanced.^53–55^ Recent studies on zwitterionic hydrogels demonstrated that mechanical training can induce structural remodeling, leading to enhanced elasticity and altered swelling responses, further highlighting the importance of network architecture in determining hydrogel properties.^56,57^

According to Peppas et al.,^58^ the diffusion exponent *n* provides insight into the physical mechanism controlling the absorption or release of a solute. Depending on the geometry of the hydrogel sample, a release exponent *n* less than 0.5 corresponds to pseudo-Fickian diffusion.^59^ When *n* equals 0.5, a classical Fickian diffusion mechanism is observed (Case I). A value of *n* = 1 indicates zero-order release kinetics (Case II transport). For 0.5 < *n* < 1, the mechanism is classified as anomalous or non-Fickian diffusion (Case III), while Super Case II transport occurs when *n* > 1. The corresponding critical *n* values for spherical particles are given in.^60^

The parameters of eq 3, calculated for the composite hydrogels studied, are presented in Table S1 (Alg hydrogels filled with stoichiometric HA) and Table S2 (those with CDHA).

The tested samples showed a general trend of decreasing *n* values with increasing CaCl_2_ concentration (i.e., degree of cross-linking). For all samples containing stoichiometric hydroxyapatite, at 0.25 wt % CaCl_2_, the swelling mechanism corresponded to the Super Case II transport (*n* > 1), indicating a polymer relaxation-controlled process. This involved expansion of the polymer matrix accompanied by conformational rearrangement of macromolecular chains and progressive pore opening. When the CaCl_2_ concentration was increased to 0.3 wt %, the swelling mechanism for all samples shifted to anomalous (non-Fickian) diffusion. At 0.5 wt % CaCl_2_, the mechanism remained anomalous, but the *n* values decreased further, suggesting a stronger contribution of water diffusion and a reduced role of polymer relaxation.

This decrease in the *n* value toward the lower boundary of the anomalous regime reflected limited polymer relaxation due to higher cross-linking density and reduced elasticity of the hydrogel network. The increasing Ca^2+^ concentration led to more egg-box structures formed by the cross-linking of G-blocks in alginate, which lowered the flexibility of the macromolecular network. Consequently, chain relaxation became restricted, limiting osmotic water uptake and favoring diffusion-dominated transport.

A similar trend of decreasing *n* with increasing filler concentration was also observed, although it was more pronounced at higher CaCl_2_ levels (0.5 wt %). This may be due to interactions between hydroxyapatite particles and the polymer matrix, which further restricted macromolecular mobility. At high filler concentrations, HA particles can act as physical barriers, reducing the free volume available for chain relaxation and water absorption. Moreover, HA particle aggregation in the hydrogel structure can lead to localized network densification, further hindering water uptake and enhancing diffusion-controlled transport.

For hydrogels filled with CDHA, these trends were less pronounced. Most of the samples synthesized with 0.25 and 0.3 wt % CaCl_2_ followed the anomalous (non-Fickian) diffusion model, where both water diffusion and polymer relaxation contribute. However, compared to the HA-filled hydrogels, the *n* values are lower, indicating a predominance of the diffusion mechanism over relaxation. CDHA contained fewer calcium ions and therefore provided fewer Ca^2+^ donors for additional cross-linking with alginate. It also exhibited different surface characteristics compared to stoichiometric HA, which may hinder strong interactions with the carboxyl groups of alginate. As a result, the hydrogel network formed was less tightly cross-linked and less elastic, allowing water to diffuse more easily without significant structural deformation, unlike in the case of HA.

For both types of hydrogels (HA and CDHA-filled), the values of the kinetic constant *k* were relatively low, ranging from 0.0051 to 0.03735 for HA and from 0.0066 to 0.1529 for CDHA, indicating slow water diffusion. A general trend of increasing *k* values with higher degrees of cross-linking (i.e., increasing CaCl_2_ concentration) was observed.

The low *k* values reflect a slow swelling rate, which was expected for ionically cross-linked hydrogels containing mineral fillers that reduced pore-free volume. The increase in *k* with cross-linker concentration may result from a higher osmotic gradient between the hydrogel and the surrounding medium due to denser network formation, which pulled water in more rapidly at the initial stages. This effect may also be influenced by the filler distribution within the HG structure.

Thus, for the studied composite hydrogels, a pronounced dependence of the swelling mechanisms on the degree of cross-linking, as well as the concentration and type of filler, was observed. At low concentrations of the cross-linking agent (*C*_CaCl_2__ = 0.25%), hydrogels filled with HA exhibited swelling behavior primarily governed by polymer relaxation. However, as the cross-linking degree increased (*C*_CaCl_2__ = 0.3% and 0.5%), water diffusion began to dominate the swelling process.

In contrast, hydrogels filled with CDHA showed a different swelling behavior compared to those with HA, with diffusion dominating over relaxation already at low cross-linker concentrations (*C*_CaCl_2__ = 0.25%). This can be attributed to the specific interaction of CDHA with alginate macromolecules, resulting in not only a less densely cross-linked but also a less relaxable, hydrogel structure.

### 3.5 Characterization of Hydrogel Sorption of Cd^2+^ Ions

Dried hydrogel materials filled with HA and CDHA (mixed with sodium alginate in the following ratios: 1:1, 1:0.5, and 1:0.25 in dry condition and 2% alginate, prepared using three concentrations of cross-linking agent—calcium chloride (0.25%, 0.3%, and 0.5%) were analyzed.

The highest sorption of Cd^2+^ ions was observed for the 2.5 g HA/2%Alg/0.25% CaCl_2_ hydrogel (157.7 mg/g) (Table S3) (sorption value was calculated for 1 g of composite powder). Generally, higher sorption ability was observed for the hydrogels with 0.25% CaCl_2_ (Figure 8). With the increase of the HA concentration, the sorption ability of hydrogels decreased. By increasing the concentration of the cross-linking agent, from 0.25% to 0.5%, the sorption of Cd^2+^ ions by the hydrogel samples was reduced. The addition of sodium alginate to stoichiometric hydroxyapatite made the sorption of Cd^2+^ ions higher compared to the pure stoichiometric hydroxyapatite. The reduction of the maximum sorption capacity of both unfilled and filled hydrogels with an increase in the cross-linking degree could be explained by the occupation of a higher amount of carboxylic groups by Ca^2+^ ions.^51^

**Figure 8:**
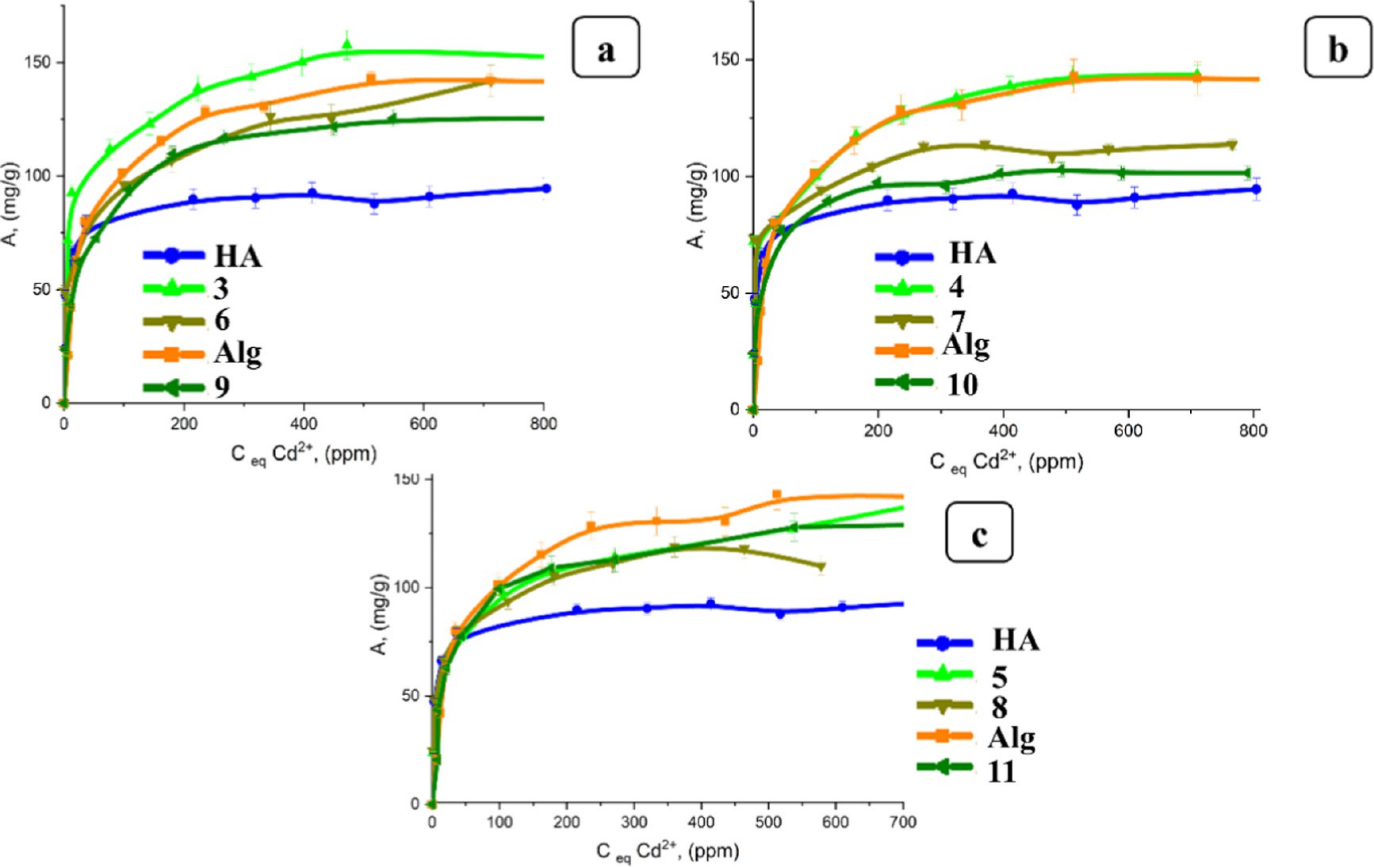
Sorption isotherms of Cd^2+^ ions by stoichiometric HA and alginate-based hydrogels filled with stoichiometric HA prepared using various concentrations of cross-linking agents: (a) 2.5 g of HA and 0.25–0.5 wt % CaCl_2_; (b) 5 g of HA and 0.25–0.5 wt % CaCl_2_; and (c) 10 g of HA and 0.25–0.5 wt % CaCl_2_.

Three mechanisms of Cd^2+^ sorption on alginate hydrogels are possible: ion exchange with Ca^2+^ ions due to the affinity of Cd^2+^ ions to the negatively charged carboxylic groups of alginate hydrogels; physical entrapment due to the porous structure of alginate hydrogels and inorganic fillers, large surface area, and Cd^2+^ ions could also be adsorbed on the hydrogel surface due to physical interactions; surface complexation of functional groups (–COO−), (–OH) on the surface of alginate hydrogels with Cd^2+^ ions.^61^ In the first step, a high rate of Cd^2+^ ion sorption was observed, and the second step was slower before reaching the equilibrium (Figure 8).

Sorption properties of hydrogels based on sodium alginate and filled with calcium-deficient hydroxyapatite, prepared using various concentrations of cross-linking agents, were also investigated (Figure 9). It was observed that hydrogels prepared using the CaCl_2_ concentrations of 0.3 and 0.25 wt % showed higher sorption values than those with 0.5 wt %. The highest sorption (190.6 mg/g) (Table S3) was observed for the hydrogel with the concentration of CaCl_2_ equal to 0.3%. Sorption ability of stoichiometric hydroxyapatite (92.5 mg/g) was higher than that for CDHA (72.13 mg/g) (Supporting Information Table S3).

**Figure 9:**
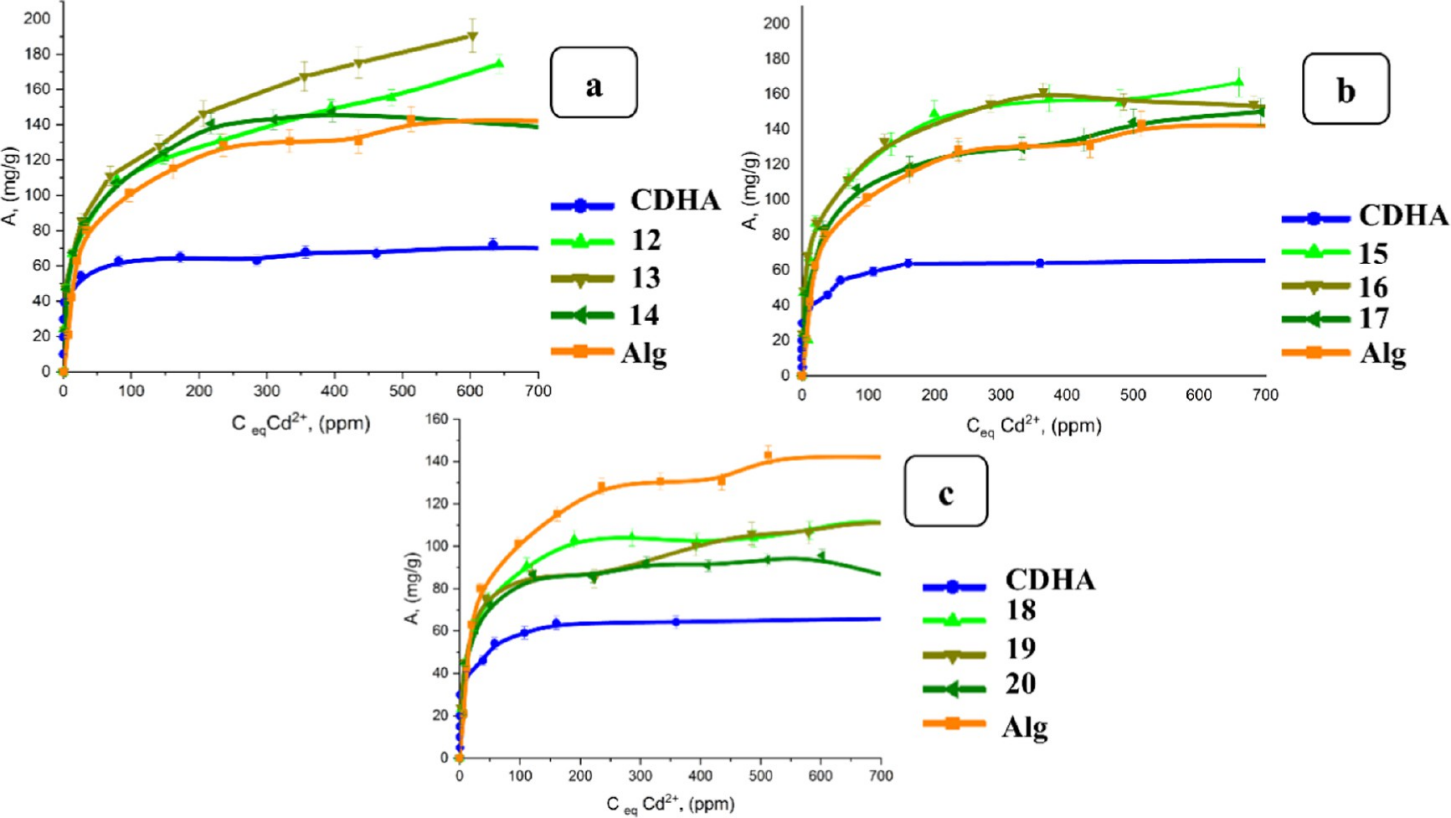
Sorption isotherms of Cd^2+^ ions by stoichiometric HA and alginate-based hydrogels filled with stoichiometric HA prepared using various concentrations of cross-linking agent: (a) 2.5 g of HA and 0.25–0.5 wt % CaCl_2_; (b) 5 g of HA and 0.25–0.5 wt % CaCl_2_; and (c) (a) 10 g of HA and 0.25–0.5 wt % CaCl_2_.

The highest sorption was observed for hydrogel with a composition of 2.5 g CDHA/2% Alg/0.3% CaCl_2_. This can be attributed to the presence of vacancies in CDHA, which were more favorable for sorption than those in HA.

The dependence of sorption properties on the mass of inorganic filler is presented in Figures 10–12. The concentration of the binding agent was kept constant for comparison to investigate the influence of different masses on hydrogel sorption. At a concentration of 0.25% CaCl_2_, the highest sorption was observed at 157.69 mg/g for 2.5 g HA/2%Alg/0.25% and 166.67 mg/g for 5 g of CDHA/2%Alg/0.25% CaCl_2._

**Figure 10:**
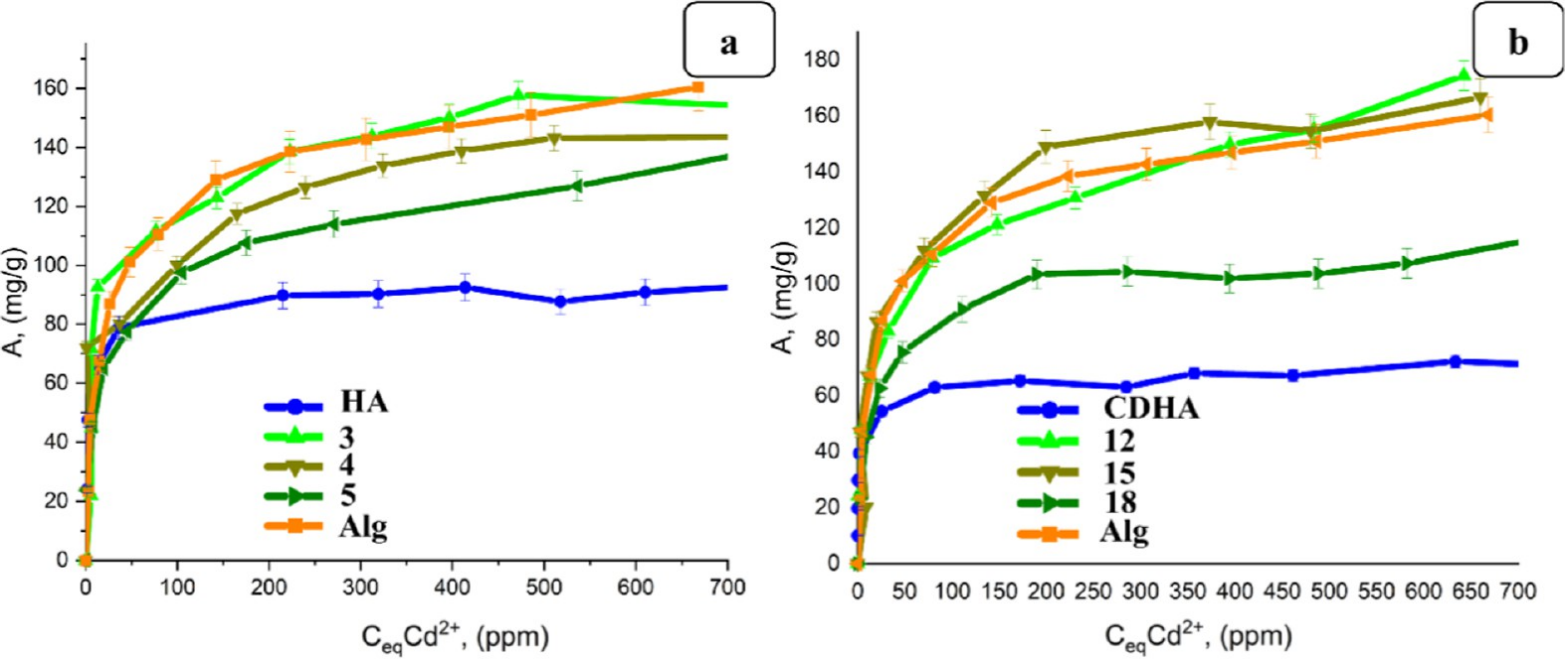
Comparison of isotherms of Cd^2+^ ion sorption by hydrogels containing (a) HA and (b) CDHA, with various masses (2.5–10 g), 2% sodium alginate and CaCl_2_ with a concentration of 0.25 wt %.

**Figure 11:**
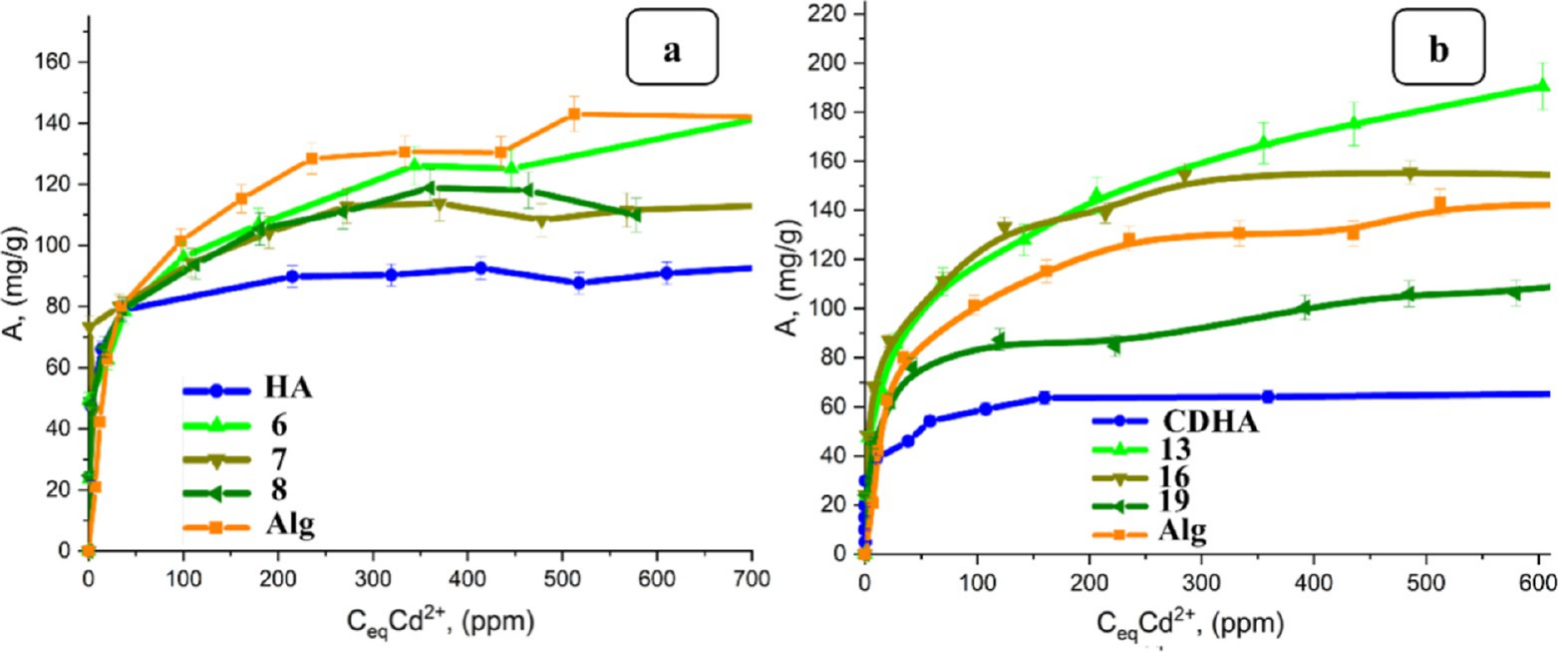
Comparison of isotherms of Cd^2+^ ion sorption by hydrogels containing (a) HA and (b) CDHA, with various masses (2.5–10 g), 2% sodium alginate and CaCl_2_ with a concentration of 0.3%.

**Figure 12:**
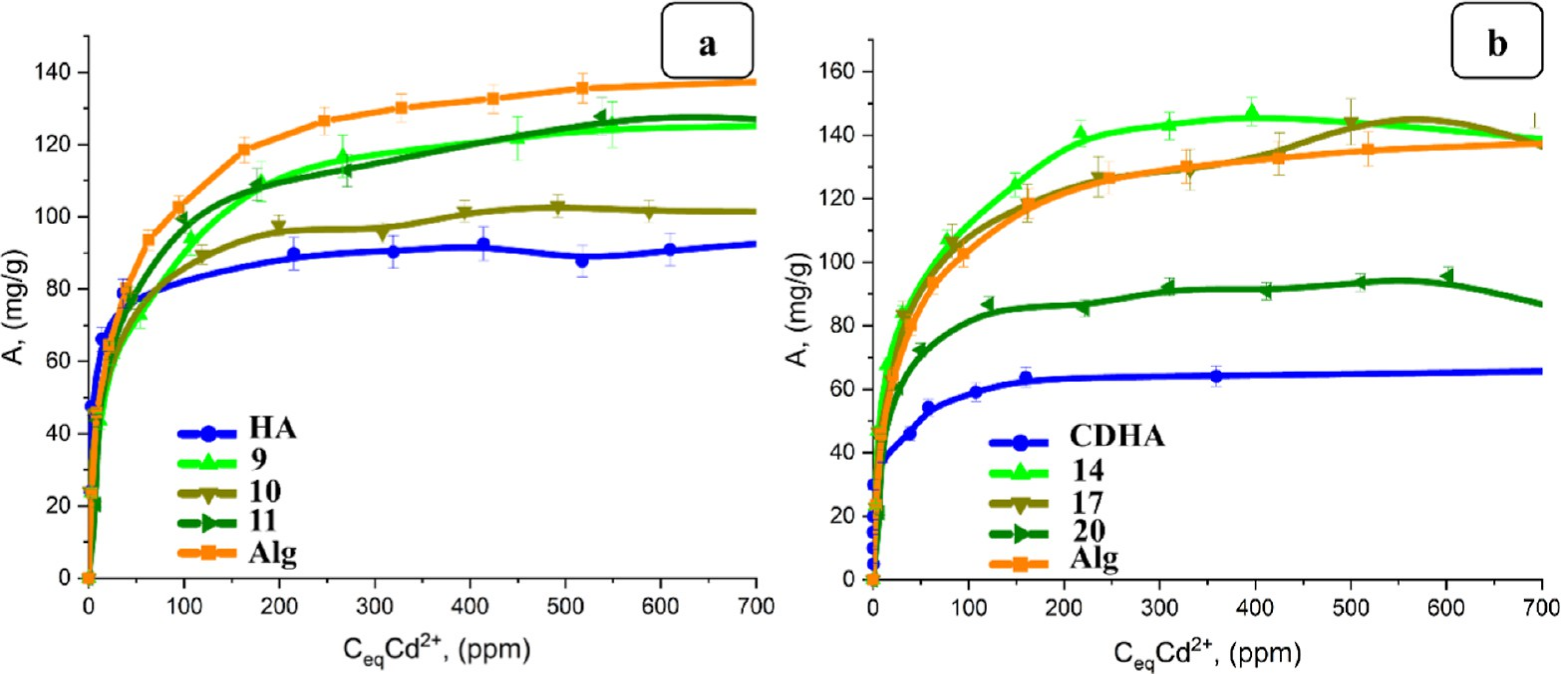
Comparison of isotherms of Cd^2+^ ion sorption by hydrogels containing (a) HA and (b) CDHA, with various masses (2.5–10 g), 2% sodium alginate and CaCl_2_ with a concentration of 0.5%.

At a concentration of 0.3% CaCl_2_, the highest sorption −141.96 mg/g was observed for 2.5 g of HA/2%Alg/0.3% and 190.58 mg/g for 2.5 g of CDHA/2%Alg/0.3% CaCl_2_ (Figure 11)_._

At the same concentration of binding agents, higher sorption was observed for the hydrogels with CDHA. With the increasing mass of inorganic fillers, the sorption decreased (Figure 11).^61^ For the hydrogels with 0.5 wt % CaCl_2_, such a dependence was not observed. Two hydrogels with masses of HA, 2.5 and 5 g, showed the highest sorption among composites, which was completely the same for both samples, but lower than for pure sodium alginate gel without filler (Figure 12a). The hydrogels filled with CDHA showed higher sorption, namely, hydrogel with composition 2.5 g of CDHA/2%Alg/0.5% CaCl_2_ sorbed a higher amount of Cd (147.38 mg/g) than pure sodium alginate gel without filler (Figure 12b).

Sorption isotherms for alginate hydrogels filled with stoichiometric and substituted HAs (synthesized at a CaCl_2_ concentration of 0.25 wt %) in the coordinates of the linear forms of the Langmuir equation, as well as the Freundlich equation, are shown in Figures S3 and S4, respectively. Additionally, Table S3 provides the matching parameters determined using the Freundlich and Langmuir models for all studied systems.

The dependence of the value of maximum sorption capacity of cadmium ions, calculated from the Langmuir equation, for the hydrogel samples prepared with the cross-linking agent concentrations of 0.25, 0.3, and 0.5 wt %, on the content of HA and CDHA is shown in Figure 13a,b, respectively.

**Figure 13:**
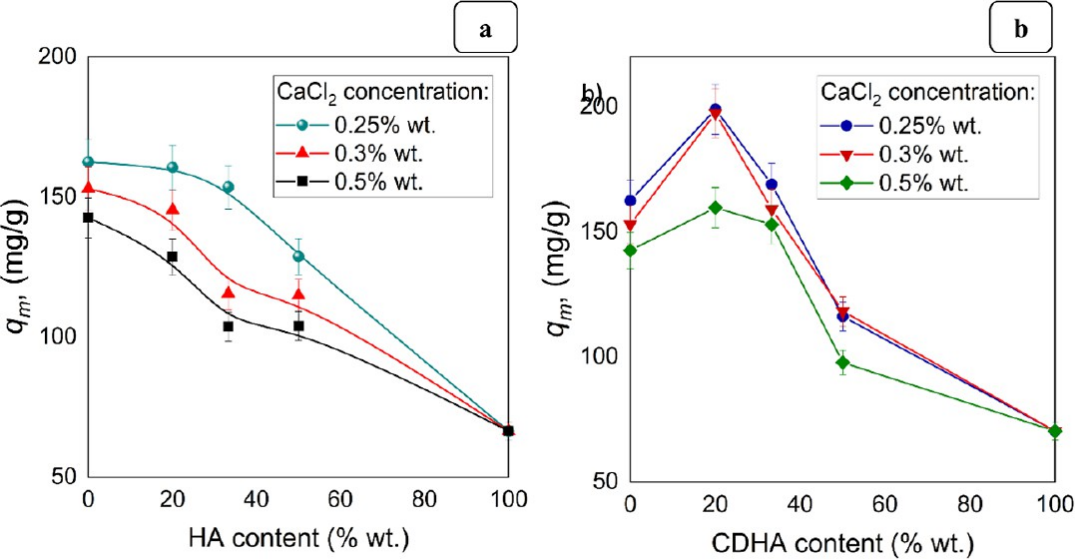
Value of the maximum sorption capacity of Cd^2+^ ions as a function of the (a) HA and (b) CDHA contents in alginate-based hydrogels. The model parameters *q*_m_ and *K*_L_ were obtained by linear regression of the *C*_e_/*q*_e_ versus *C*_e_ plot using the least-squares method. The goodness of fit was evaluated based on the correlation coefficient *R* ^2^.

As can be seen from,^62^ the Ca/P relation in apatites influenced the sorption properties of various chemical compounds. Our results illustrated that higher sorption was observed for hydrogels containing CDHA, which could be explained by the existence of substitutions in the crystal structure leading to the incorporation of other ions.

### 3.6 Characterization of Hydrogel Biosafety

The viability of pea seeds after contact with the hydrogels was assessed using Nelyubov’s method (Figure 14).

**Figure 14:**
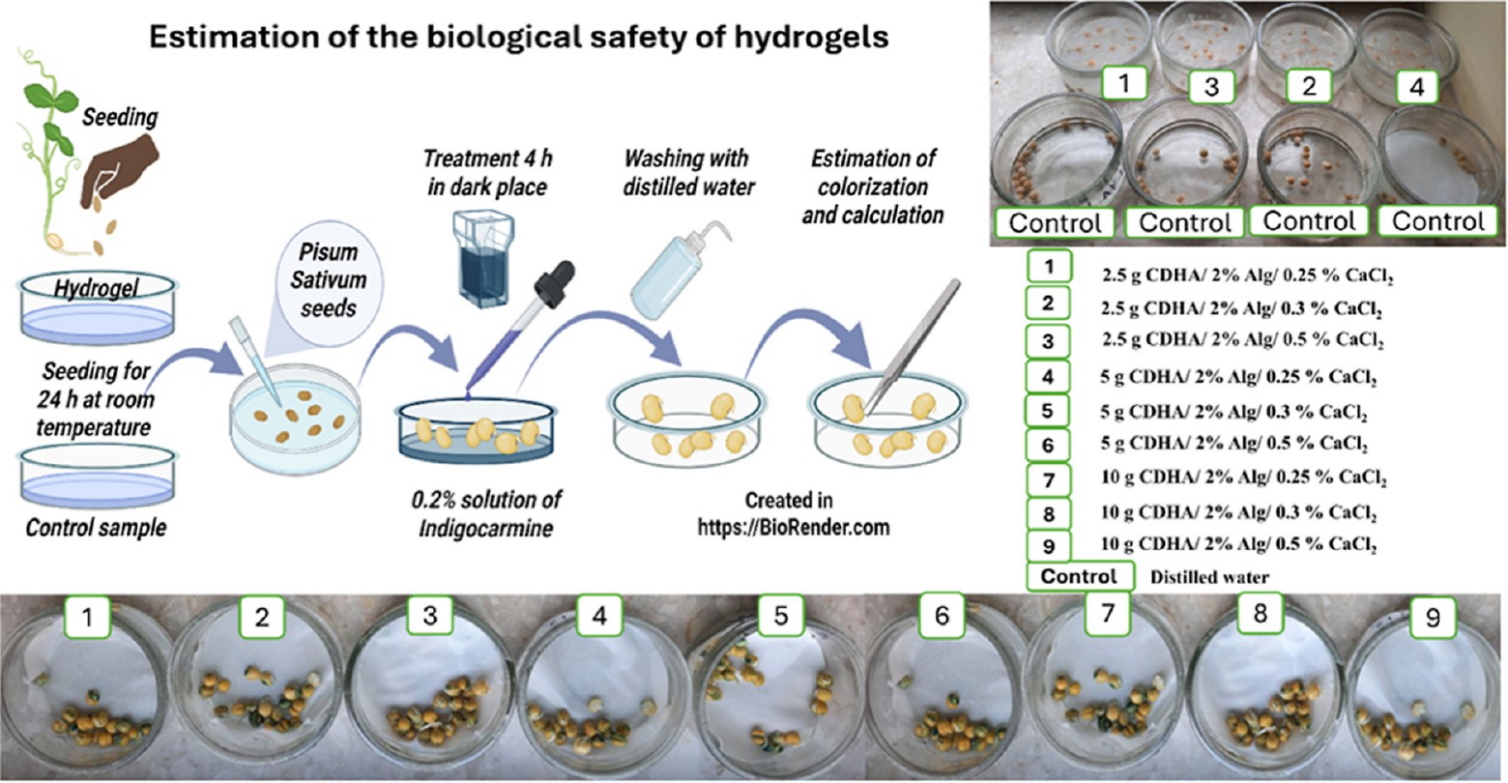
Testing of biocompatibility of the synthesized hydrogels by Nelyubov’s method.

Embryos of *P. sativum* after indigo carmine colorization were rinsed with water and examined for staining: embryos with slightly partially stained cotyledons or completely unstained were considered viable. Seed viability (percentage of viable seeds) is presented in Table S4, allowing assessment of the hydrogel ecotoxicity. The assay revealed almost no notable differences in seed viability depending on the composition of the hydrogel samples. For most cases, the viability equaled 95 ± 5% (*n* = 3). The control group (seeds incubated in distilled water) exhibited high viability, with the same average of 95 ± 5% (*n* = 3) unstained or partially stained embryos, indicating minimal cellular damage. These findings suggest that HA/Alg- and CDHA/Alg-based hydrogels are nontoxic under the tested conditions and show potential for agricultural applications. The minor decline in viability (to the value of 90 ± 5%) observed in some variants indicates the need for further optimization of hydrogel composition to minimize potential phytotoxic effects.

The *L. sativum* seed germination test provided additional insight into the biological compatibility and functional impact of the hydrogels. The germination percentage in the control group was 100% (*n* = 3), with an average root length of 2.6 cm. The description of seed germination experiments is shown in Figure 15.

**Figure 15:**
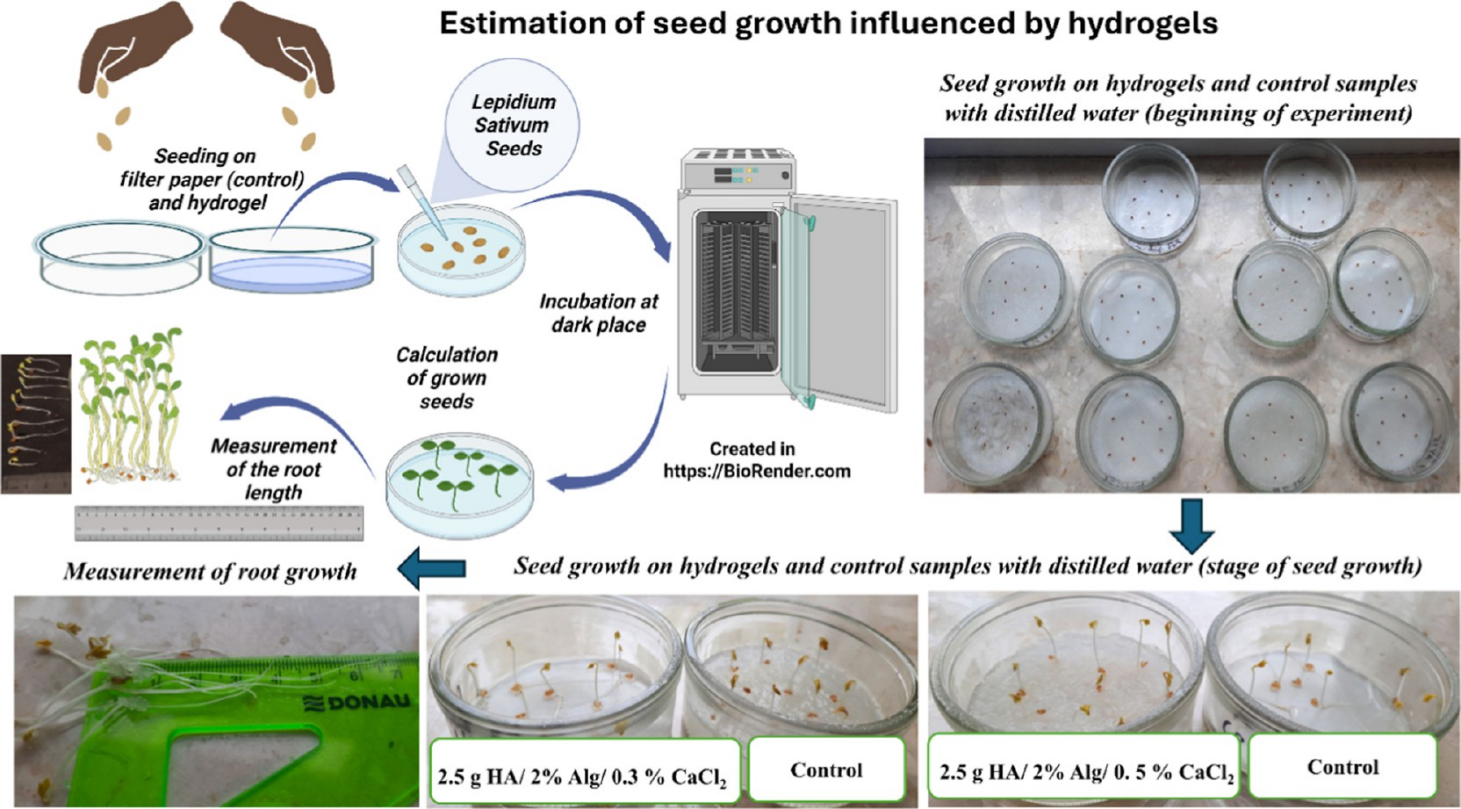
Determination of Seed Growth and Root Growth of *Lepidium Sativum* on hydrogel samples in comparison with control (distilled water).

The results of the percentage of germination—RSG, root length—relative root growth (RRG) and germination index (GI) are presented in Table S5.

Hydrogels with stoichiometric HA maintained a high germination rate (RSG = 80–100%) and promoted root elongation, resulting in an RRG increase from 117.72 to 193.02%, nearly twice that of the control. This suggested hydrogels based on stoichiometric HA were nontoxic and stimulated root growth. This stimulation can be associated with favorable water retention properties of the hydrogel matrix, as well as the potential release of bioavailable elements from the hydroxyapatite phase and alginate-derived oligomeric fragments that can exert biostimulatory effects. The HA-filled hydrogels significantly enhanced seed performance relative to the control, achieving GI = 105.95–193.02% and RRG 117.72–193.02%, which can be attributed to the controlled release of Ca^2+^ and PO_4_^3–^ supporting early metabolic activity and root development. Conversely, the hydrogels with CDHA showed a slight inhibitory effect. GI ranged from 77.56% to 139.80%, and RRG from 86.18 to 155.4%. This meant that a decrease by almost 14% occurred in some samples (compared to the control), indicating a mild inhibitory effect likely arising from its higher solubility and rapid ion release, leading to localized ionic or osmotic stress. The physicochemical properties of the CDHA/Alg hydrogel may temporarily limit root expansion, which can be associated with differences in the network structure and water–polymer interactions within the hydrogel matrix. The results of pH measurements for HA and CDHA and their hydrogels after 1 h and 6 days of water immersion illustrated that the initial pH was changed from 7.01 to 7.22 after 6 days for HA and from 6.98 to 7.94 for CDHA. For all hydrogels, pH was at the range from 6.4 to 7.4 after 6 days corresponding to neutral medium.

Thus, the differing crystallinity and dissolution behavior of HA and CDHA govern nutrient availability and microenvironmental stability. It is very important to adjust the composition of the apatite phase to optimize the plant growth response. The obtained results also demonstrated that the developed hydroxyapatite–alginate hydrogels, particularly those based on stoichiometric HA, were biologically safe and biocompatible for use in agriculture. Their ability to maintain or enhance seed viability and to stimulate early root growth supports their application as functional soil conditioners. Additionally, their ability to retain moisture makes them promising tools for sustainable soil management, especially in drought-prone regions. The less biocompatibility observed in formulations with CDHA highlighted the need to balance hydrogel composition, as changes in calcium/phosphate ratios can affect the ionic environment around seeds.

## 4 Conclusions

Composite hydrogels based on sodium alginate and hydroxyapatite were developed and comprehensively characterized, with potential relevance for environmentally related applications. It was found that the swelling capacity was primarily determined by cross-linker concentration. The hydrogels with 0.25% CaCl_2_ exhibited the highest swelling, while those with 0.5% showed minimal water uptake. The type of hydroxyapatite filler strongly influenced swelling mechanisms. The HA-filled hydrogels demonstrated polymer relaxation-dominated behavior at low cross-linking, whereas the CDHA-filled ones were governed by diffusion even under mild cross-linking conditions. The lowest swelling ability was observed in hydrogels with the highest concentration of cross-linking agent, 0.5 wt % CaCl_2_. With a decrease in the binding agent concentration to 0.25 wt %, the swelling of the obtained hydrogels increased. The amount of inorganic filler in the composite hydrogel did not have a significant influence on the swelling properties of the composite materials. Both HA and CDHA enhanced cadmium sorption compared to pure alginate, with maximum sorption capacities of 157.7 mg/g (HA) and 190.6 mg/g (CDHA), respectively. The sorption process fitted best to the Langmuir isotherm model.

Biosafety assessments confirmed that the developed composites were nontoxic to plants. Hydrogels with hydroxyapatite not only maintained but also stimulated seed germination and root elongation, supporting their potential as biocompatible materials for soil-related applications.

The obtained results suggested that the developed materials combine water retention, pollutant immobilization, and plant growth support, thus representing a potentially promising approach for soil remediation and agricultural applications. However, these conclusions are based on laboratory-scale experiments, and further studies are required to assess long-term environmental behavior, including biodegradability, ecotoxicity, and life-cycle aspects. The future research should focus on long-term soil incubation and field-scale experiments to evaluate cadmium immobilization under realistic environmental conditions, as well as to assess agronomic performance across different soil types. These investigations will build directly on the current laboratory findings by addressing scale-related limitations and verifying the stability, durability, and real-world applicability of the proposed materials.

## Supplementary Material



## Data Availability

The experimental data that support the findings of this study are openly available in RepOD: https://repod.icm.edu.pl/dataset.xhtml?persistentId=doi:10.18150/JM49WQ.

## Notes

Description of X-ray diffraction method, swelling studies, measurements of sorption of Cd^2+^ by hydrogels, as well as the results of X-ray diffraction, SEM imaging, hydrogel swelling, sorption data modeling, and biosafety assay (PDF)
