# Supplementary material for: Development of Eco-Friendly Hydrogels Loaded with Stoichiometric and Calcium-Deficient Hydroxyapatites for Sustainable Agriculture
Source: ACS Appl Mater Interfaces. 2026 Apr 28;18(18):26934–51. doi: 10.1021/acsami.6c03612 (PMC13181726; doi:10.1021/acsami.6c03612)
Supplement: Supplementary file 1 [file am6c03612_si_001.pdf]

Supporting information

Development of eco-friendly hydrogels loaded with stoichiometric and calcium-deficient hydroxyapatites for sustainable agriculture

Ganna Yanovska[1,2], Olena Goncharuk[1,3], Dmytro Honcharuk[3,4], Nataliia Guzenko[1,5], Ewa Skwarek[6], Katarzyna Szewczuk-Karpisz[1]*

[1] Institute of Agrophysics, Polish Academy of Sciences, Doświadczalna 4, 20-290 Lublin, Poland

[2] Sumy State University, Kharkivska Str., 2, 40007, Sumy, Ukraine

[3] F.D. Ovcharenko Institute of Biocolloidal Chemistry, NAS of Ukraine, 42 Acad. Vernadskoho Ave., 03142 Kyiv, Ukraine

[4] Taras Shevchenko National University Academician Glushkov Avenue, 4, Kyiv, 03680 Ukraine

[5] Chuiko Institute of Surface Chemistry NAS of Ukraine 17, General Naumov's Street, 03164, Kyiv, Ukraine

[6] Maria Curie-Skłodowska University, M. Curie-Skłodowskiej Sq. 5, 20-031, Lublin, Poland

*corresponding author: k.szewczuk-karpisz@ipan.lublin.pl

2. Materials and Methods

2.3. Inorganic fillers characterization

2.3.1. X-Ray diffraction

X-ray diffraction measurements were conducted using a diffractometer configured in Bragg–Brentano vertical geometry, with a goniometer radius of 142 mm. The scan range spanned from +3° to 154° in 2θ, with a variable scan speed ranging from 0.0001° to 100°/min (2θ). The system offered an angular precision of ±0.02° 2θ and a step resolution of 0.0003°, with a peak broadening of less than 0.05° 2θ. The X-ray radiation source was a sharp-focus copper tube with a metal-ceramic construction, rated at 1500 W (PROTO). A high-voltage generator operating at 30 kV and 20 mA (600 W total) powered the X-ray source. Diffraction data were acquired using a DECTRIS® MYTHEN 2R one-dimensional hybrid detector equipped with 640 channels.

2.4.3. Swelling studies

For the experiment, a sample of dried hydrogel (0.015-0.02 g) was weighed with an accuracy of four digits and placed in glass jars. 10 ml of distilled water was added and left to swell. At controlled intervals, HGs samples were filtered using a fine sieve. and residual moisture was carefully removed with a lint-free paper filter. In the next step, the HGs were weighed. Then they returned to jars with 10 ml of distilled water for further swelling. After which the experiment was repeated until an equilibrium state was reached, when the mass of the sample remained constant. To study the kinetics of swelling and the mechanism of solvent diffusion into HGs. All experiments were carried out at least three times. The experimental data were presented as mean values, with standard deviations that did not exceed 10 % of their mean values in all tests.

2.4.4. Sorption of Cadmium by Hydrogels

The study of the Cd(II) ions sorption was provided in the following way: the sorbed amount of Cd(II) ions on hydrogels was estimated by the difference of their concentration in the studied solution before and after the sorption process. Sorption isotherms were measured at the concentration of cadmium ions equal to 50, 100, 150, 200, 300, 400, 500, 600, 800, and 1000 mg/L.

At the beginning, the required amount of the solid substance was placed in a test tube, and the solution of cadmium ions with an initial concentration of 1000 ppm at the appropriate dilution was added. Such prepared systems were shaking at a speed of 20 rpm. To define the sorption isotherms, the shaking process was carried out for 48 h. After the end of sorption, the solid substance was separated by centrifugation at 3000 rpm for 5 min (centrifuge SBS-LZ-4000/20-6, Steinberg Systems), and the

concentration of Cd was determined with an ion-selective electrode (detector S.A. Electrochemical Equipment, the sensitivity range of the electrode from 10^{-7} - 0.1 M) in the obtained clear solutions.

3. Results and discussion

3.1. X-Ray diffraction of inorganic fillers

Phase composition of as-prepared powders of inorganic fillers corresponding to hydroxyapatite (JCPDS card 04-021-1904).

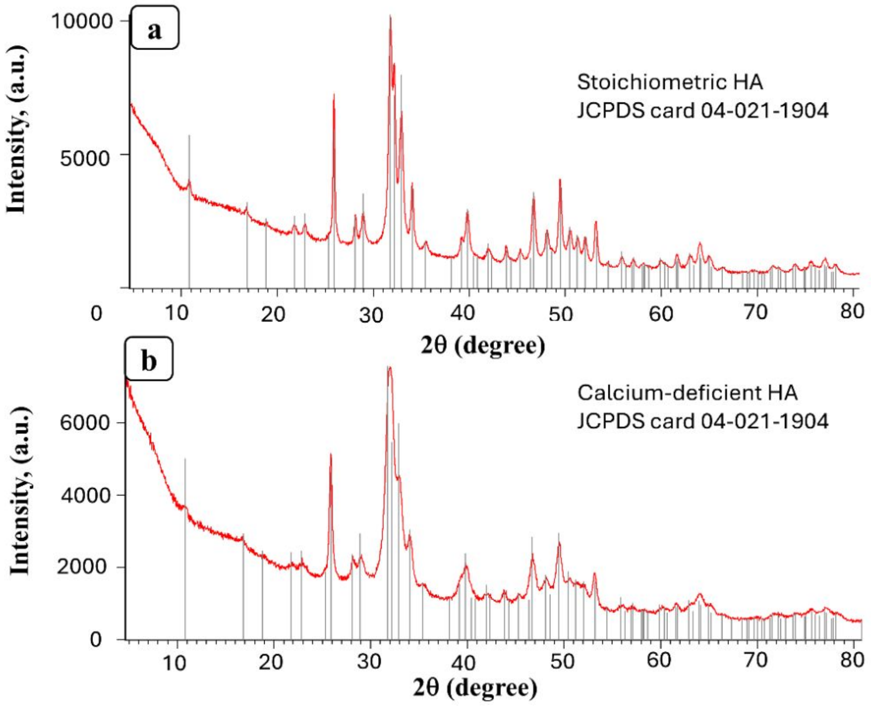

Figure S1. XRD patterns of a) stoichiometric HA, b) calcium-deficient CDHA.

3.2. Scanning electron microscopy

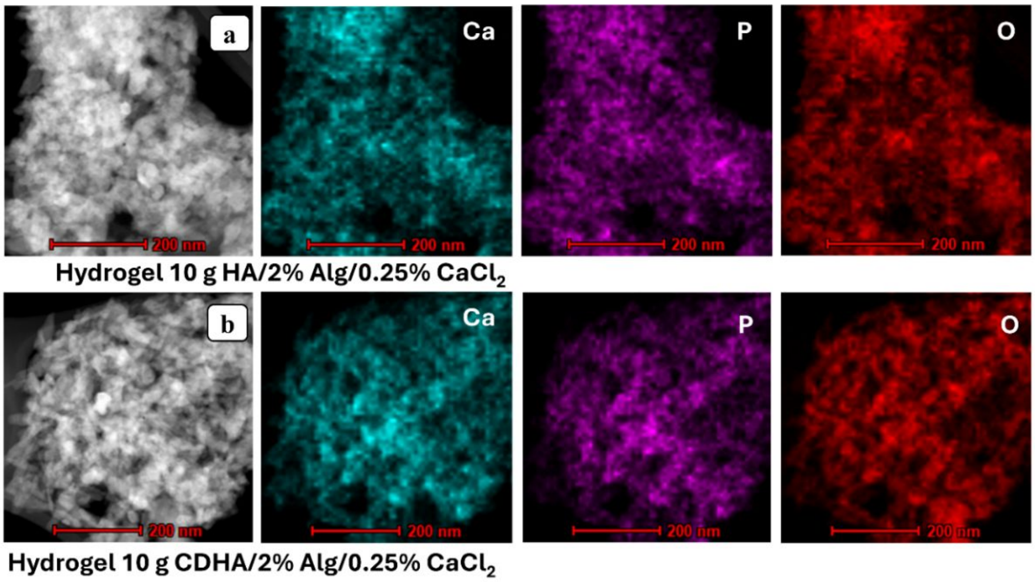

Figure S2. SEM image of hydrogel 5 (a) and hydrogel 18 with EDX analysis

The SEM images combined with EDX elemental mapping confirmed the successful incorporation of hydroxyapatite particles within the alginate hydrogel matrix. In both hydrogel samples, calcium (Ca), phosphorus (P), and oxygen (O) were homogeneously distributed throughout the structure, indicating uniform dispersion of the mineral phase in the polymeric network. For the hydrogel containing stoichiometric HA (sample a), the EDX maps revealed a relatively dense and compact distribution of Ca and P, consistent with the TEM observations of aggregated HA nanocrystals embedded in the alginate matrix. In contrast, the hydrogel with calcium-deficient hydroxyapatite (sample b) exhibited a more diffuse and less compact elemental distribution, reflecting the lower crystallinity and smaller particle size of CDHA. These differences in elemental distribution patterns suggested that HA provided a more stable and well-ordered mineral phase within the hydrogel. At the same time, CDHA contributed to a more porous and open structure, potentially resulting in higher surface reactivity. The uniform presence of oxygen across both samples additionally confirmed the contribution of the alginate matrix and the mineralized hydroxyapatite phase.

3.3. Estimation of hydrogel swelling properties

Table S1. Composition of the studied samples and parameters of swelling kinetics of the HGs based on Alg filled with 10 g stoichiometric HA.

Sample code	Filler content C_{filler}. wt%	Alg content. wt%	*C* $CaCl_2$. wt%	*n*	*k*	R^2	Type of diffusion
3	20.0	80.0	0.25	1.16	0.0085	0.9793	Super case II transport
4	33.3	66.7	0.25	1.09	0.0084	0.9823	Super case II transport
5	50.0	50.0	0.25	1.15	0.0051	0.9835	Super case II transport
6	20.0	80.0	0.30	0.93	0.0146	0.9758	anomalous (non-Fickian) diffusion
7	33.3	66.7	0.30	0.989	0.0201	0.9624	Case II diffusion
8	50.0	50.0	0.30	0.756	0.0286	0.9994	anomalous (non-Fickian) diffusion
9	20.0	80.0	0.50	1.02	0.0086	0.9843	Case II diffusion
10	33.3	66.7	0.50	0.799	0.0250	0.9691	anomalous (non-Fickian) diffusion
11	50.0	50.0	0.50	0.684	0.0374	0.8886	anomalous (non-Fickian) diffusion

Table S2. Composition of the studied samples and parameters of swelling kinetics of the HGs based on Alg filled with substituted HA.

**Sample code**	**Filler content. wt%**	**Alg content. wt%**	**C $CaCl_2$. Wt %**	***n***	***k***	R^2	**Type of diffusion**
12	20	80	0.25	0.5289	0.1228	0.9625	anomalous (non-Fickian) diffusion
13	20	80	0.30	0.8658	0.04072	0.9734	anomalous (non-Fickian) diffusion
14	20	80	0.50	0.3241	0.1529	0.9209	Fickian diffusion
15	33.3	66.7	0.25	1.2790	0.0066	0.96519	anomalous (non-Fickian) diffusion
16	33.3	66.7	0.30	0.9547	0.0181	0.9927	anomalous (non-Fickian) diffusion
17	33.3	66.7	0.50	0.60461	0.0966	0.9546	anomalous (non-Fickian) diffusion

18	50	50	0.25	0.5404	0.08624	0.98430	anomalous (non-Fickian) diffusion
19	50	50	0.30	0.8262	0.02582	0.9695	anomalous (non-Fickian) diffusion
20	50	50	0.50	0.6175	0.05398	0.9812	anomalous (non-Fickian) diffusion

3.4. Characterization of sorption of Cd^{2+} ions on hydrogels

The values of the Freundlich sorption intensity factor (1/n) were in the range from 0.1 to 0.3, which indicated a favourable sorption process for all Alg/HA hydrogels. The coefficients of determination (R^2) calculated from the linear regression for the selected models showed that the Langmuir isotherm monolayer coating model provided a better fit to the experimental data than the empirical Freundlich model describing sorption on heterogeneous surfaces. Nevertheless, the trends in the influence of both fillers (HA and CDHA), as well as the concentration of the cross-linking agent on the maximum sorption capacity (monolayer saturation q_m), calculated using the Langmuir model, and on the Freundlich sorption capacity (K_F) generally remain very similar for all the studied materials.

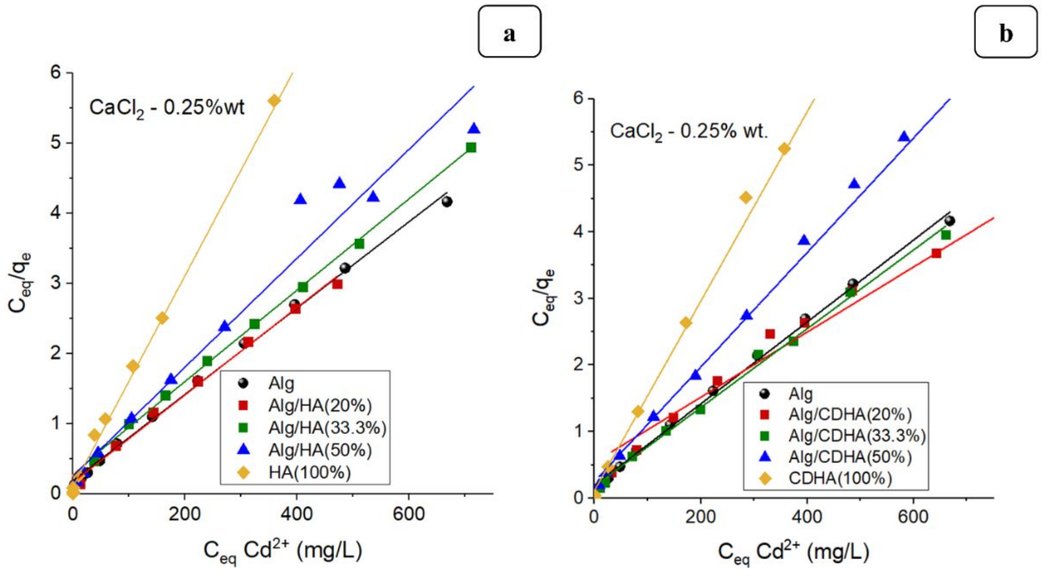

Figure S3. Langmuir isotherm plots for the alginate hydrogels filled with HA (a) and CDHA synthesized using the $CaCl_2$ concentration of 0.25% wt. (b).

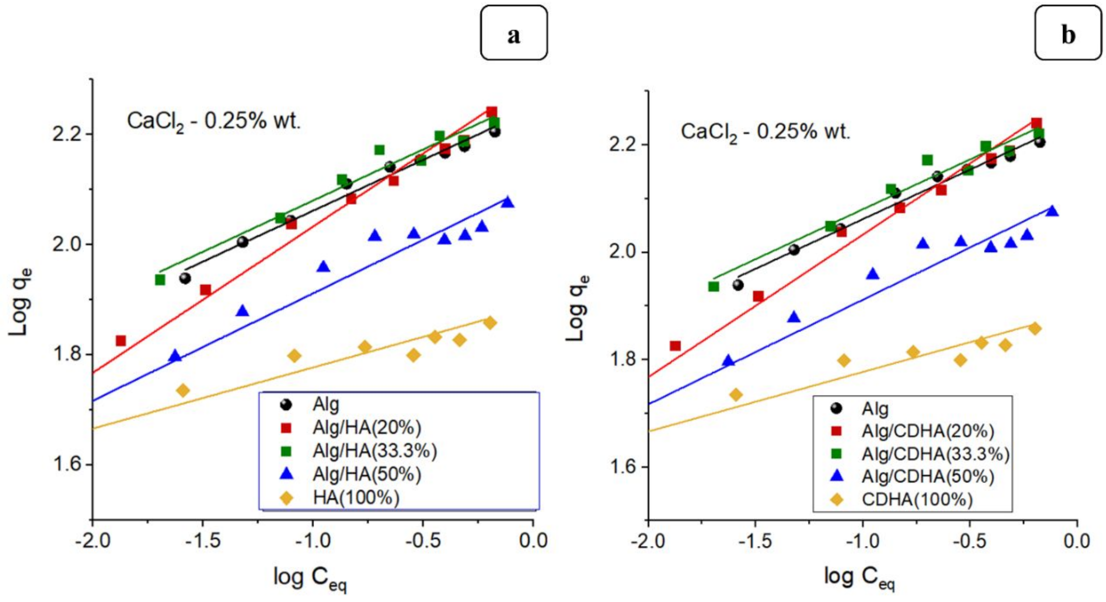

Figure S4. Freundlich isotherm plots for the alginate hydrogels filled with HA (a) and CDHA synthesized using the $CaCl_2$ concentration of 0.25% wt. (b).

3.5. Characterization of hydrogel biosafety

Table S4. The results of Nelubov's testing for HA and CDHA inorganic fillers and the hydrogels of various compositions (SD = 5%, n = 3).

Sample composition	Seeds Viability (%)
HA	95
CDHA	95
2.5 g HA/ 2% Alg/ 0.25 % $CaCl_2$	90
2.5 g HA/ 2% Alg/ 0.3 % $CaCl_2$	95
2.5 g HA/ 2% Alg/ 0.5 % $CaCl_2$	95
2.5 g CDHA/ 2% Alg/ 0.25 % $CaCl_2$	95
2.5 g CDHA/ 2% Alg/ 0.3 % $CaCl_2$	90
2.5 g CDHA/ 2% Alg/ 0.5 % $CaCl_2$	95
5 g HA/ 2% Alg/ 0.25 % $CaCl_2$	95
5 g HA/ 2% Alg/ 0.3 % $CaCl_2$	95
5 g HA/ 2% Alg/ 0.5 % $CaCl_2$	95
5 g CDHA/ 2% Alg/ 0.25 % $CaCl_2$	95
5 g CDHA/ 2% Alg/ 0.3 % $CaCl_2$	90

5 g CDHA/ 2% Alg/ 0.5 % $CaCl_2$	95
10 g HA/ 2% Alg/ 0.25 % $CaCl_2$	90
10 g HA/ 2% Alg/ 0.3 % $CaCl_2$	100
10 g HA/ 2% Alg/ 0.5 % $CaCl_2$	100
10 g CDHA/ 2% Alg/ 0.25 % $CaCl_2$	100
10 g CDHA/ 2% Alg/ 0.3 % $CaCl_2$	100
10 g CDHA/ 2% Alg/ 0.5 % $CaCl_2$	100

Table S5. The results of the germination tests for HA and CDHA inorganic fillers and the hydrogels of various compositions (SD = 5%, n = 3).

Sample composition	RSG (%)	RRG (%)	GI (%)
HA	90	172.36	155.13
CDHA	80	145.60	116.50
2.5 g HA/ 2% Alg/ 0.25 % $CaCl_2$	90	117.72	105.95
2.5 g HA/ 2% Alg/ 0.3 % $CaCl_2$	100	120.17	120.17
2.5 g HA/ 2% Alg/ 0.5 % $CaCl_2$	80	130.07	104.06
2.5 g CDHA/ 2% Alg/ 0.25 % $CaCl_2$	90	90.60	81.54
2.5 g CDHA/ 2% Alg/ 0.3 % $CaCl_2$	100	126.70	126.70
2.5 g CDHA/ 2% Alg/ 0.5 % $CaCl_2$	90	155.40	139.80
5 g HA/ 2% Alg/ 0.25 % $CaCl_2$	100	110.97	110.97
5 g HA/ 2% Alg/ 0.3 % $CaCl_2$	100	142.32	142.32
5 g HA/ 2% Alg/ 0.5 % $CaCl_2$	100	192.64	192.64
5 g CDHA/ 2% Alg/ 0.25 % $CaCl_2$	100	119.70	119.70
5 g CDHA/ 2% Alg/ 0.3 % $CaCl_2$	80	106.65	85.32
5 g CDHA/ 2% Alg/ 0.5 % $CaCl_2$	100	87.31	87.31
10 g HA/ 2% Alg/ 0.25 % $CaCl_2$	100	168.61	168.61
10 g HA/ 2% Alg/ 0.3 % $CaCl_2$	100	176.12	176.12
10 g HA/ 2% Alg/ 0.5 % $CaCl_2$	100	193.02	193.02
10 g CDHA/ 2% Alg/ 0.25 % $CaCl_2$	90	86.18	77.56
10 g CDHA/ 2% Alg/ 0.3 % $CaCl_2$	90	107.96	97.16
10 g CDHA/ 2% Alg/ 0.5 % $CaCl_2$	100	102.33	102.33
